# A High-Phosphorus Diet Moderately Alters the Lipidome and Transcriptome in the Skeletal Muscle of Adult Mice

**DOI:** 10.3390/nu15173734

**Published:** 2023-08-25

**Authors:** Sarah M. Grundmann, Kerstin Ress, Lea Zimmermann, Marcus Höring, Gerhard Liebisch, Erika Most, Robert Ringseis, Klaus Eder

**Affiliations:** 1 Institute of Animal Nutrition and Nutrition Physiology, Justus Liebig University Giessen, Heinrich-Buff-Ring 26-32, 35392 Giessen, Germany; kerstin.fackelmann@ernaehrung.uni-giessen.de (K.R.); lea.zimmermann@ernaehrung.uni-giessen.de (L.Z.); erika.most@ernaehrung.uni-giessen.de (E.M.); robert.ringseis@ernaehrung.uni-giessen.de (R.R.); klaus.eder@ernaehrung.uni-giessen.de (K.E.); 2 Institute of Clinical Chemistry and Laboratory Medicine, University Hospital of Regensburg, Franz-Josef-Strauss-Allee 11, 93053 Regensburg, Germany; marcus.hoering@ukr.de (M.H.); gerhard.liebisch@ukr.de (G.L.); 3 Center for Sustainable Food Systems, Justus Liebig University Giessen, Senckenbergstrasse 3, 35390 Giessen, Germany

**Keywords:** high phosphorus, skeletal muscle, lipidome, transcriptome, β-oxidation, mice

## Abstract

A high phosphorus intake has been associated with various metabolic disorders, including chronic kidney disease, cardiovascular disease, and osteoporosis. Recent studies have demonstrated the effects of dietary phosphorus on lipid and glucose metabolism. This study investigated the impact of a high-phosphorus diet on mouse skeletal muscle lipid composition and gene transcription. Adult male mice (*n* = 12/group) received either a diet with an adequate (0.3%) or a high (1.2%) phosphorus concentration for 6 weeks. The lipidome analysis showed that among the 17 analyzed lipid classes, the concentrations of three classes were reduced in the high phosphorus group compared to the adequate phosphorus group. These classes were phosphatidylethanolamine (PE), phosphatidylglycerol (PG), and lysophosphatidylcholine (LPC) (*p* < 0.05). Out of the three hundred and twenty-three individual lipid species analyzed, forty-nine showed reduced concentrations, while three showed increased concentrations in the high phosphorus group compared to the adequate phosphorus group. The muscle transcriptome analysis identified 142 up- and 222 down-regulated transcripts in the high phosphorus group compared to the adequate phosphorus group. Gene set enrichment analysis identified that genes that were up-regulated in the high phosphorus group were linked to the gene ontology terms “mitochondria” and “Notch signaling pathway”, whereas genes that were down-regulated were linked to the “PI3K-AKT pathway”. Overall, the effects of the high-phosphorus diet on the muscle lipidome and transcriptome were relatively modest, but consistently indicated an impact on lipid metabolism.

## 1. Introduction

It has been estimated that the daily phosphorus intake across Western industrialized nations is two to three times [1,2,3] higher than the recommended amounts of 700 mg/day for adults [4,5,6,7]. Phosphorus is generally present as phosphate in inorganic and organic compounds. Phosphates are commonly used as additives in processed foods [8], for example as preservatives, acidifiers, acidity regulators, release agents, and emulsifiers. It is estimated that between 40% and 70% of the top-selling foods and beverages, including cola drinks, frozen foods, dry mixes, packaged meat, breads, and baked goods, contain phosphorus additives [9]. Animal products, such as milk and milk products (21.0%) and meat and meat products (25.2%), are the main sources of dietary phosphorus intake, but it can also be present in cereal products (29.3%) [10].

Phosphorus is an essential mineral in nutrition and an essential component of the bone matrix, cell membranes, DNA, and RNA, as well as energy-transferring molecules, like ATP. Phosphorus plays a key role in bone metabolism, and an excess intake of phosphorus can lead to abnormal bone mineralization and decreased bone density [6]. However, studies of the last decades have shown that a dietary phosphorus intake has far more influences on the metabolism than the well-known effects on the skeleton. A high intake of phosphorus has been linked to a range of metabolic disorders, including chronic kidney disease, cardiovascular disease, and osteoporosis [11,12,13]. In addition, a high phosphorus intake can also disrupt calcium metabolism, which can contribute to the development of cardiovascular disease and other health problems [14].

Recent studies have demonstrated the effects of dietary phosphorus on lipid and glucose metabolism [15,16,17]. Rats fed a high-phosphorus diet had lower body weights, lower amounts of visceral fat, lower levels of plasma insulin and non-esterified fatty acids, and a lower respiratory exchange ratio. Additionally, the high-phosphorus diet suppressed the transcription of lipogenic genes (e.g., *Srebf1*, *Fas*, and *Acaca*) in the liver. The high-phosphorus diet also resulted in an increase in the gene transcription of uncoupling protein 1 and peroxisome proliferator-activated receptor-γ coactivator-1α (*Ppargc1a*) in brown adipose tissue [18]. Transcriptome analysis of the liver of rats fed a high-phosphorus diet showed that the high level of phosphorus led to a down-regulation of genes involved in hepatic amino acid catabolism and lipogenesis, while genes related to the β-oxidation of fatty acids were up-regulated. Ingenuity pathway analysis suggested that PPARα was activated in the livers of rats that were fed a high-phosphorus diet. Moreover, the serum concentration of fibroblast growth factor 21 (FGF21), which promotes fatty acid utilization in adipose tissue as a PPARα target gene, was higher in rats that were fed the high-phosphorus diet than in those that were fed the control diet [16]. The association between phosphorus and glucose is believed to be mediated by insulin [19], as a result of its capacity to stimulate both phosphorus uptake by the muscles [20] and phosphorylation of many compounds, thereby creating a competition [21] among these compounds for phosphorus.

Lipids have a variety of functions in the organism, including their contribution to the structural integrity of cell membranes, insulation and protection of vital organs, hormone synthesis, and vitamin absorption and transport, but they also serve as an energy source. The process of lipid metabolism involves the breakdown of triacylglycerols, which are stored in adipose tissue, into glycerol and fatty acids. Glycerol can be converted into glucose via gluconeogenesis, while fatty acids are transported to the mitochondria where they undergo β-oxidation to produce acetyl-CoA. These processes result in the production of ATP, which provides energy for energy-consuming reactions. Studies have suggested that dietary phosphorus can affect the rate of ATP production [22,23,24]. Additionally, dietary phosphorus significantly affects the activity of several enzymes involved in energy metabolism, including pyruvate kinase and phosphoenolpyruvate carboxykinase [25]. Furthermore, the availability of dietary phosphorus affects the activity of hormones involved in energy metabolism, such as insulin and glucagon [26].

Influences of a high-phosphorus diet on lipid and energy metabolism can, in turn, be relevant for skeletal muscle, as this tissue represents one of the primary energy consumers of the body. During endurance exercise, when glycogen reverses are depleted, fatty acids become an essential source of energy for muscle tissue. Despite the potential connection between a high intake of phosphorus and muscle metabolism, few studies have examined the influence of a high-phosphorus diet on skeletal muscle metabolism. Peri-Okonny et al. [27] conducted a study on the effects of a high-phosphorus diet in mice. In this study, the high phosphorus group showed exercise intolerance and impaired fatty acid metabolism compared to the control mice. Specifically, the high phosphorus group had a lower exercise capacity, reduced oxygen consumption, and decreased fatty acid oxidation during exercise. The authors suggested that these effects may be due to alterations in mitochondrial function and impaired oxygen delivery to muscles in mice that were fed the high-phosphorus diet. Further studies have shown effects of a high-phosphorus diet on muscle gene transcription and composition [17,27]. These effects included the down-regulation of genes involved in fatty acid release and oxidation, including *Fabp4*, *Hsl*, *Fasn*, and *Pparg* [27]. Furthermore, the high-phosphorus diet has been associated with increased activation of the mammalian target of rapamycin (mTOR) pathway and extracellular-signal regulated kinases (Erks) in the muscles of aged mice, as observed in the study conducted by Ugrica et al. [17]. mTOR plays a crucial role in regulating protein synthesis, a process vital for cell growth and maintenance, whereas Erk signaling is associated with fiber-type switching in the muscle.

Overall, these findings suggested that a high-phosphorus diet has significant effects on skeletal muscle metabolism, particularly impacting concentrations of lipids and genes involved in fatty acid metabolism and signaling pathways related to muscle growth and protein synthesis. However, due to the limited number of studies and scarcity of data on this topic, further investigations are needed. This study investigated the hypothesis that a high-phosphorus diet alters skeletal muscle lipid composition and gene transcription, potentially affecting skeletal muscle metabolism and fiber type transformation. To this end, explorative lipidome and transcriptome analyses were performed in the skeletal muscle of adult mice that were fed an adequate or high-phosphorus diet for a period of 6 weeks.

## 2. Materials and Methods

### 2.1. Animals and Diets

The animal experiment was carried out according to the German animal welfare law and was declared to the Animal Welfare Officer of the university (Registration No.: M_721, approval date: 6 August 2019). The animal husbandry facility was licensed by the local authorities (Az: FD 62 §11 JLU Stoffwl). The methods used for the humane euthanasia of the animals were in accordance with the recommendations of the AVMA guidelines for the euthanasia of animals.

The experiment included 24 male, 19-week-old C57BL/6J mice purchased from Charles River (Sulzfeld, Germany). Animals were maintained in groups of two under controlled conditions (12 h light: 12 h dark, 22 ± 1 °C ambient temperature, and 50–60% relative humidity). After an adjustment period of one week, mice were randomly assigned to two groups (adequate phosphorus (P_0.3_) and high phosphorus (P_1.2_)) of 12 mice each. The two groups received semi-synthetic diets (Table 1), which met the requirements of mice according to the National Research Council (NRC), for a period of 6 weeks [6]. The diet of the adequate phosphorus group contained 3 g of phosphorus per kg (0.3%; P_0.3_); the diet of the high phosphorus group contained 12 g of phosphorus per kg (1.2%; P_1.2_). The concentration in the high phosphorus group was three times the recommendation for mice. This concentration was chosen based on findings from other studies with mice and rats, which demonstrated significant effects on lipid metabolism in the liver [16] and muscle [27] at this particular phosphorus concentration. The source of phosphorus in both diets was potassium dihydrogen phosphate (KH_2_PO_4_). The diet with adequate phosphorus concentrations contained potassium bicarbonate (KHCO_3_) to achieve equal concentrations of potassium in both diets.

The adequate- and high-phosphorus diets contained 16.6 MJ and 16.5 MJ gross energy/kg DM, respectively, and the analyzed phosphorus concentrations of the two diets were 2.89 and 11.9 g/kg DM, respectively. All diets were fed for 6 weeks. The mice had free access to diet and water. Diet intake and body weight were measured weekly.

### 2.2. Sample Collection

Mice were decapitated under CO_2_ anesthesia. Blood was collected in heparin-coated tubes. After centrifugation (10,000× *g*, 10 min) at 4 °C, plasma was collected. Livers, hearts, and kidneys were removed, washed in ice-cold NaCl solution (0.9%), dried, weighed, and several small aliquots were collected and frozen in liquid nitrogen. In addition, several muscles (*musculus rectus femoris*, *musculus gastrocnemius*, and *musculus soleus*) were collected, weighed, and frozen. All samples were stored at −80 °C until analysis.

### 2.3. Determination of the Apparent Total Tract Digestibility of Nutrients and Energy

Apparent total tract digestibility (ATTDs) of nutrients (calcium, phosphorus, crude fat, and crude ash) and energy were determined using the indicator method. After fecal samples were collected form each cage, they were freeze dried and ground using a centrifugal mill (Retsch, Haan, Germany). In these fecal samples, the concentration of the indigestible indicator titanium dioxide (TiO_2_) was determined according to the slightly modified method of Brandt and Allam [28]. Fecal concentrations of crude fat, crude ash, and energy were determined according to the official VDLUFA methods (VDLUFA 2007). Based on the concentrations of the indicator, nutrients, and energy in the diet and feces, respectively, the ATTDs of the nutrients and energy were calculated, according to Hafez et al. [29], with Formula (1).

(1) $$ATTD\left(\%\right)=100-\left[\left(\frac{{TiO}_{2_{Diet}}}{{TiO}_{2_{Feces}}}\right)\cdot \left(\frac{{Nutrient}_{Feces}}{N{utrient}_{Diet}}\right)\cdot 100\right]$$

### 2.4. Determination of the Plasma Concentrations of Calcium and Inorganic Phosphate

The plasma concentrations of calcium and inorganic phosphate were measured spectrophotometrically according to the manufacturer’s instructions (Fluitest^®^ CA and Fluitest^®^ PHOS, Analyticon Biotechnologies AG, Lichtenfels, Germany).

### 2.5. Determination of the Concentrations of the Major Lipid Classes and Individual Phospholipid Species by Means of Lipidomics

Lipidomics analysis was employed to analyze the composition of lipids in the muscle tissue. The analysis was performed according to the methods already described [30,31]. In brief, the lipids of 2 mg muscle homogenates were extracted using the Bligh and Dyer method [32]. For this process, internal standards were added (PC 14:0/14:0, PC 22:0/22:0, PE 14:0/14:0, PE 20:0/20:0 (di-phytanoyl), PS 14:0/14:0, PS 20:0/20:0 (di-phytanoyl), PI 17:0/17:0, LPC 13:0, LPC 19:0, LPE 13:0, Cer d18:1/14:0, Cer 17:0, D7-FC, CE 17:0, and CE 22:0). The chloroform phase was collected, vacuum dried, and the residues were dissolved in either 10 mmol/L ammonium acetate in methanol/chloroform (3:1, *v*/*v*; for low-resolution tandem mass spectrometry) or in chloroform/methanol/2-propanol containing 7.5 mmol/L ammonium formate (1:2:4 *v*/*v*/*v*; for high-resolution mass spectrometry).

The extracts were subjected to direct flow injection (FIA) analysis using either a triple quadrupole mass spectrometer (FIA-MS/MS; QQQ triple quadrupole) or a hybrid quadrupole orbitrap mass spectrometer (FIA-FTMS; high mass resolution). FIA-MS/MS (QQQ) was performed in positive ion mode, as previously described [33,34]. For LPC, a fragment ion of *m/z* 184 was used [35]. For PE, LPE, PS, PG, and PI, the following neutral losses were used: 141, 141, 185, 189, and 277, respectively [36]. PE P were analyzed according to the principles described by Zemski Berry et al. [37]. With a fragment ion of *m/z* 264, sphingosine-based Cer 18:1; O2 and HexCer 18:1; O2 were analyzed [38]. Lipid species were annotated according to Liebisch et al. [39]. For these data, the annotation of glycerophospholipid species was based on the assumption that only even-numbered carbon chains were present.

The Fourier transform mass spectrometry (FIA-FTMS) setup was described previously [40]. TG and CE were acquired in positive ion mode FTMS in the *m/z* range 500–1000 for 1 min with a maximum injection time (IT) of 200 msec, an automatic gain control (AGC) of 1 × 106, three microscans, and a target resolution of 140,000 (at 200 *m/z*). PC and PC O were measured in negative ion mode in the *m/z* range of 520–960. For FC, multiplexed acquisition (MSX) was used, as previously described [40]. ALEX software 2017 was used for data processing [41]. The extracted data were exported to and further processed using Microsoft Excel 2010 with self-programmed macros. Due to the moderate effect of the high-phosphorus diet on the muscle lipidome, application of the false discovery rate (FDR)-adjusted *p*-value threshold of <0.05 did not reveal statistically significant lipid species. Therefore, lipid classes and individual lipid species, as well as the relative distribution of lipid species within their lipid class, were considered as significantly different if the statistical analysis resulted in a raw *p*-value of <0.05.

### 2.6. Extratction of Total RNA

Frozen muscle aliquots (40–50 mg) were used to extract total RNA using TRIzol reagent (Invitrogen, Karlsruhe, Germany), following the manufacturer’s instructions. The RNA samples’ quantity and quality were assessed using an Infinite 200 M microplate reader with a NanoQuant plate (both from Tecan, Mainz, Germany). The average A260/A280 ratio of all total RNA samples was 1.90 ± 0.04 (*n* = 24).

### 2.7. Microarray and Bioinformatic Analysis

Six randomly selected samples of total muscle RNA from each group were used for microarray analysis. The RNA quality was assessed based on A260:A280 ratios and RNA integrity number (RIN) values, which were 1.84 ± 0.07 (mean ± standard deviation) and 7.48 ± 0.31, respectively, following the method described by Gessner et al. [42]. The RNA samples were processed at the Genomics Core Facility, “KFB—Kompetenzzentrum für Fluoreszente Bioanalytik” (Regensburg, Germany); according to the manufacturer’s protocol (Applied Biosystems GeneChip Whole Transcript (WT) PLUS Reagent Kit User Guide, Thermo Fisher Scientific, Waltham, MA, USA). Briefly, total RNA (200 ng) was used to generate double-stranded cDNA. The cRNA (12 µg) was purified and reverse-transcribed into single-stranded (ss) cDNA, incorporating unnatural dUTP residues.

To prepare for hybridization, the purified ss cDNA was fragmented using a combination of uracil DNA glycosylase (UDG) and apurinic/apyrimidinic endonuclease 1 (APE 1), and then labeled with biotin. The processed ss cDNA (3.8 µg) was hybridized to the gene chip (GeneChip Clariom S mouse arrays, Applied Biosystems, Thermo Fisher Scientific) for 16 h at 45 °C and 60 rpm in a hybridization oven (GeneChip hybridization oven 640, Applied Biosystems). The hybridized arrays were washed and stained (GeneChip Fluidics Station FS450, Applied Biosystems), and fluorescence signals were measured (GeneChip Scanner 3000 7G system, Applied Biosystems) using the GeneChip Command Console v4.3.3 software (Applied Biosystems) to control the fluidics and scanning functions.

The GCCN-SST-RMA algorithm (GeneChip Expression Console v1.4 software, Applied Biosystems) was used to calculate summarized probe set signals under a log_2_ scale. Average signal values, comparative fold changes (FCs), and significant *p*-values were calculated using Microsoft Excel. MIAME-compatible microarray data were deposited into the NCBI public repository Gene Expression Omnibus ([43]; GEO accession number GSE 236311).

Similar to the lipidome data, feeding a high-phosphorus diet compared to the adequate phosphorus resulted in only modest changes in the transcriptome. Consequently, applying the FDR *p*-value threshold of <0.05 led to the identification of a limited count of significantly changed transcripts. Unfortunately, this count proved to be insufficient for conducting a gene set enrichment analysis (GSEA). As an alternative approach, raw *p*-values were adopted using a significance threshold of 0.05 to address this limitation. Therefore, transcripts with an FC > 1.3 and a Student’s *t*-test raw *p*-value < 0.05 were defined as up-regulated, while those with an FC < −1.3 and a Student’s *t*-test raw *p*-value < 0.05 were defined as down-regulated. Similar filtering criteria have been employed in recent studies [44,45,46] where the transcriptomic changes caused by the intervention were also moderate. Consequently, the differentially expressed transcripts were subjected to GSEA to identify enriched gene ontology (GO) terms using the Database for Annotation, Visualization, and Integrated Discovery (DAVID) 6.8 [47,48]. GO terms with *p* < 0.05 were considered enriched. Separate GSEA analyses were conducted for the transcripts that were up-regulated and those that were down-regulated.

### 2.8. qPCR Analysis to Validate Microarray Data and Assess mRNA Abundance

For the validation of microarray data and the assessment of the mRNA abundance of genes related to myosin heavy chain isoforms and energy metabolism, qPCR analysis was performed. A total of twelve differentially expressed transcripts were selected for the microarray validation. In general, total RNA samples of each mouse (*n* = 12/group) were utilized for the qPCR analysis. The cDNA was synthesized following a recently described protocol [47]. The qPCR analysis was performed using gene-specific primer pairs obtained from Eurofins MWG Operon (Ebersberg, Germany), as previously described [47]. The primers were designed using the Basic Local Alignment Search Tool (BLAST) and Primer3. The detailed primer properties can be found in the Supplementary Materials (Table S1). For data normalization, the method described by Vandesompele et al. [48] was applied using the three most stable reference genes (actin, beta, *Actb*; beta-2 microglobulin, *B2m*; and peptidylprolyl isomerase A, *Ppia*) out of the five potential reference genes tested.

### 2.9. Western Blot Analysis

Western blot analysis was performed following the protocol described by Zeitz et al. [49]. In summary, frozen muscle tissue (30 mg) was homogenized in RIPA buffer containing protease and phosphatase inhibitor solutions. After centrifugation, the supernatants were stored at −20 °C. Concentrations of proteins were determined using the BC assay method (UP40840A, Interchim, Montlucon, France).

For separation via SDS-PAGE, 30 µg of proteins were loaded onto the SDS gel using 4% separation gel and 10% running gel. A molecular weight marker (10–170 kD, PageRuler prestained protein ladder; Thermo Scientific, Dreieich, Germany) was included in each gel. The proteins were then electro-transferred for 1.5 h at 100 V and 300 mA onto a nitrocellulose membrane (Pall, Pensacola, FL, USA) using a blotting buffer with 5% methanol. Following Ponceau S (Carl Roth, Karlsruhe, Germany) staining to verify equal loading, the membranes were blocked using 5% skimmed milk powder (for protein kinase B (AKT), phosphorylated AKT (pAKT), and vinculin) or 5% BSA (for forkhead box O1 (FoxO1) and phosphorylated FoxO1 (pFoxO1)) in TBST.

The following primary antibodies were used for the detection of AKT, pAKT, FoxO1, and pFoxO1: Akt (pan) (11E7) Rabbit mAb #4685 from Cell Signaling Technology Europe B.V. (CST), Frankfurt, Germany; Phospho-Akt (Ser473) (D9E) XP^®^ Rabbit mAb #4060 from CST; FoxO1 (L27) Antibody #9454 from CST; Phospho-FoxO1 (Ser256) Antibody #9461 from CST; and Vinculin Recombinant Rabbit Monoclonal Antibody (42H89L44) from Invitrogen. Primary antibodies were diluted 1:1000 in the blocking buffer and the membranes were incubated with the primary antibody solution overnight at 4 °C (for pAKT, FoxO1, pFoxO1, and vinculin) or for 2 h at 20 °C (for AKT). After washing, a horseradish peroxidase-conjugated secondary polyclonal goat anti-rabbit IgG antibody (A0545, Sigma Aldrich, Taufkirchen, Germany) was added to the membranes and incubated for 1.5 h at room temperature, diluted at a ratio of 1:10,000 in TBST. After washing, the blots were developed using the ECL Select™ western blotting detection reagent (GE Healthcare, Munich, Germany) for 5 min at room temperature. The intensities of the specific bands were detected using a Bio-Imaging system (G:BOX, Syngene, Cambridge, UK) and quantified using Syngene GeneTools 4.3.1 software. Unfortunately, the presence of unspecified binding in the antibodies for AKT, pAKT, FoxO1, and pFoxO1 resulted in the appearance of double bands upon detection. This occurrence prompted us to assign the upper band to phosphorylated proteins, as phosphorylation often brings about a subtle augmentation in protein mass. Correspondingly, we allocated the lower band to non-phosphorylated proteins. This procedure has already been described in the literature [50].

### 2.10. Principal Component Analysis of the Lipidome Data

Data preparation and principal component analysis (PCA) of the muscle lipidome data were performed using the MetaboAnalystR 3.2 package for R version 4.2.1 [51]. For PCA, the relative metabolite composition of each individual lipid species within their different lipid classes was used. Prior to the PCA, variables with missing values were either excluded from the analyses (if more than 50% of the samples were missing) or replaced by LoDs (1/5 of the minimum positive value of each variable). After normalization through log transformation and autoscaling, the remaining values were used for the PCA.

### 2.11. Statistical Analysis

Statistical analyses were performed using SPSS 27 software (IBM, Armonk, NY, USA). The cage served as the experimental unit for feed and energy intake, all ATTD parameters, and the individual animal for all other data. The Shapiro–Wilk test was used to test all parameters for normal distribution and Levene’s test was used to test for homoscedasticity. Logarithmic transformed data were used for statistical analysis when normal distribution was only observed after being logarithmized. Differences between groups were assessed using different statistical tests based on the characteristics of the data. If the data met the criteria for normal distribution and had homogeneous variances, the Student’s *t*-test was used. If the variances were found to be heterogeneous, the Welch’s *t*-test was applied. In cases where the data did not follow a normal distribution, the Mann–Whitney U test was used. For all statistical tests, a *p*-value less than 0.05 was considered to be statistically significant.

## 3. Results

### 3.1. Growth Performance and Organ Weights

Differences in body weight between the two groups were observed from the 5th week of feeding, with P_1.2_ mice having a significantly lower body weight than the P_0.3_ mice (*p* < 0.05, Table 2). Feed and gross energy intakes showed no significant differences between the two groups (*p >* 0.05, Table 2). Tibial length, as a marker of body size, was not significantly different between the two groups of mice (*p >* 0.05, Table 2). No differences were found in organ weights of the heart, kidneys, *M. soleus*, *M. gastrocnemius,* or *M. rectus femoris* (*p >* 0.05, Table 2) between the two groups. Liver weights were significantly lower in the P_1.2_ group compared to the P_0.3_ group (*p* < 0.05, Table 2).

### 3.2. Apparent Total Tract Digestibility of Phosphorus, Calcium, Crude Ash, Crude Lipids, and Gross Energy

The ATTD of phosphorus was 1.8 times higher in the P_1.2_ group than in the P_0.3_ group (*p* < 0.05, Table 3). The ATTD of crude lipids was also higher in the P_1.2_ group than in the P_0.3_ group (*p* < 0.05, Table 3). The ATTD of crude ash and gross energy were slightly lower in the P_1.2_ group than in the P_0.3_ group (*p* < 0.05, Table 3), while the ATTD of calcium did not significantly differ between the two groups.

### 3.3. Plasma Concentrations of Calcium and Inorganic Phosphate

In order to characterize the mineral status of the mice, serum concentrations of calcium and inorganic phosphate were analyzed. P_1.2_ mice had significantly lower plasma concentrations of calcium than P_0.3_ mice (*p* < 0.05, Table 4). Plasma phosphorus concentrations were not found to be significantly different between the two groups (*p* > 0.05, Table 4).

### 3.4. Effect of a High-Phosphorus Diet on the Muscle Lipidome

**Figure 1:**
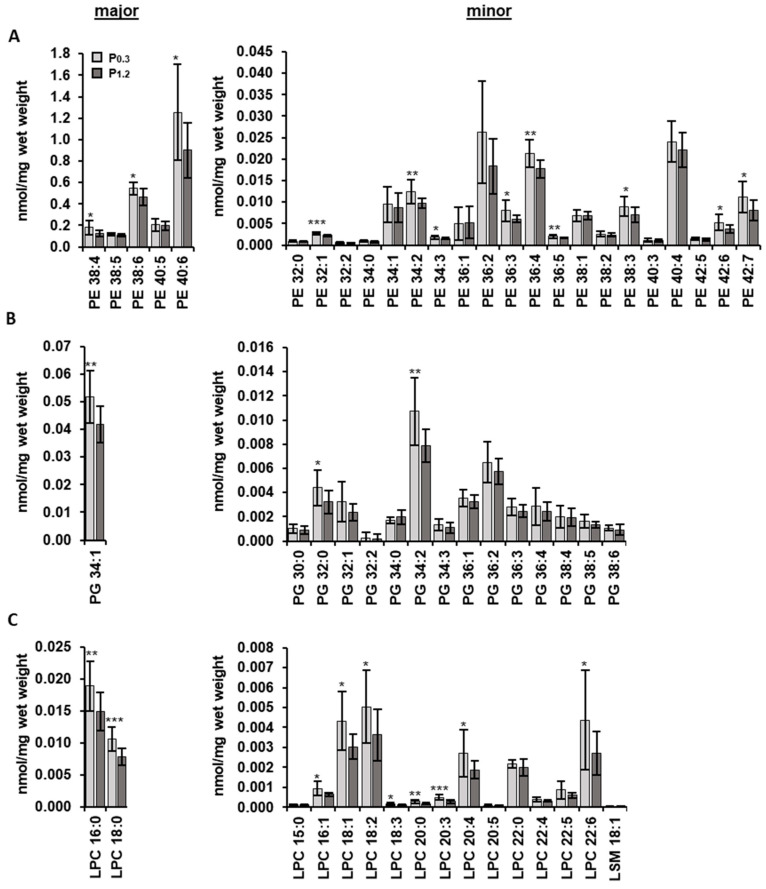
Concentrations of individual species of phosphatidylethanolamine (PE) (**A**), phosphatidylglycerol (PG) (**B**), and lysophosphatidylcholine (LPC) (**C**) (including lysosphingomyelin (LSM)) in the *M. gastrocnemius* of adult male C57BL/6J mice. Mice were fed a diet containing either 0.3% (P_0.3_) or 1.2% (P_1.2_) phosphorus for 6 weeks. Data are mean values and standard deviations, *n* = 12 mice/group. * (*p <* 0.05), ** (*p* < 0.01), and *** (*p* < 0.001) indicate statistically significant differences.

Lipidomic analysis of *M. gastrocnemius* was performed. A total of 323 molecules from 17 lipid classes were characterized. The concentrations of phosphatidylethanolamine (PE), phosphatidylglycerol (PG), and lysophosphatidylcholine (LPC) were all lower in the P_1.2_ group compared to the P_0.3_ group (*p* < 0.05, Table 5), while the remaining lipid classes including, triacylglycerol and free cholesterol, were unchanged between the two groups. Out of the 323 individual lipid species analyzed, the concentration of 52 individual lipid species were significantly influenced in response to the phosphorus concentrations in the diet (Table S2). Fifty percent of the regulated species were minor species with concentrations below a 0.01 nmol/mg wet weight. With the exception of three individual lipid species, all other species affected showed lower concentrations in the P_1.2_ group than in the P_0.3_ group. Out of the 24 PE species analyzed, 12 showed reduced concentrations in the P_1.2_ group compared to the P_0.3_ group (*p* < 0.05, Figure 1A). Similarly, out of the 15 PG species analyzed, three showed lower concentrations in the P_1.2_ group than in the P_0.3_ group (*p* < 0.05, Figure 1B). For the 16 LPC species that were analyzed, 10 displayed reduced concentrations in the P_1.2_ group compared to the P_0.3_ group (*p* < 0.05, Figure 1C). The lipid composition, expressed as the percentages of individual lipids related to total lipids, was not significantly different between the two groups (*p* > 0.05, Table 5).

**Figure 2:**
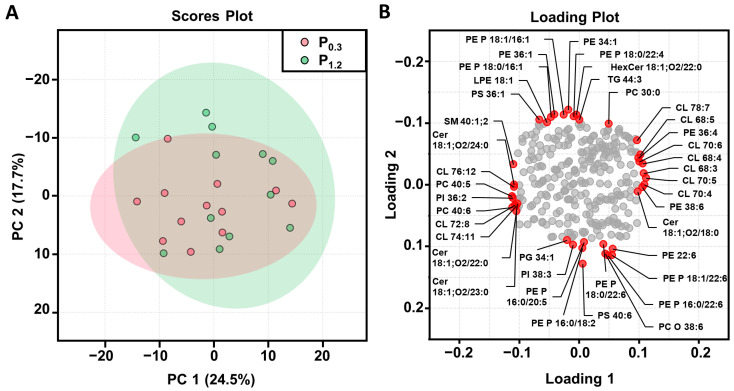
Principal component analysis (PCA) of the lipidome of the *M. Gastrocnemius* of adult male C57BL/6J mice. Shown are the scores plot (**A**) with a plotted 5% confidence interval and the corresponding loading plot (**B**) of the PCA. Mice were fed a diet containing either 0.3% (P_0.3_) or 1.2% (P_1.2_) phosphorus for 6 weeks. The data represent the principal components (PC 1 or PC 2) and their loadings that were calculated on the basis of the relative abundances of the individual lipid species within their respective lipid classes, *n* = 12 mice/group. Abbreviations: cardiolipin (CL), ceramide (Cer), hexosylceramide (HexCer), lysophosphatidylethanolamine (LPE), phosphatidylcholine (PC), PC ether (PC O), phosphatidylethanolamine (PE), PE-based plasmalogens (PE P), phosphatidylglycerol (PG), phosphatidylinositol (PI), phosphatidylserine (PS), sphingomyelin (SM), and triacylglycerols (TG).

To identify any patterns or relationships among the measured variables, a principal component analysis (PCA) was carried out. The PCA plot illustrates the distribution of the 24 mice in our dataset based on two principal components that together explained 42.2% of the variance of the dataset. The scores plot displayed no clustering, indicating that there was no clear separation of the samples based on the measured variables (Figure 2A). In accordance, the loading plot (Figure 2B) showed the contribution of each lipid species to the principal component and confirmed that no specific lipid species was a main driver of separation.

### 3.5. Transcripts in the Muscle Regulated through the High-Phosphorus Diet

**Figure 3:**
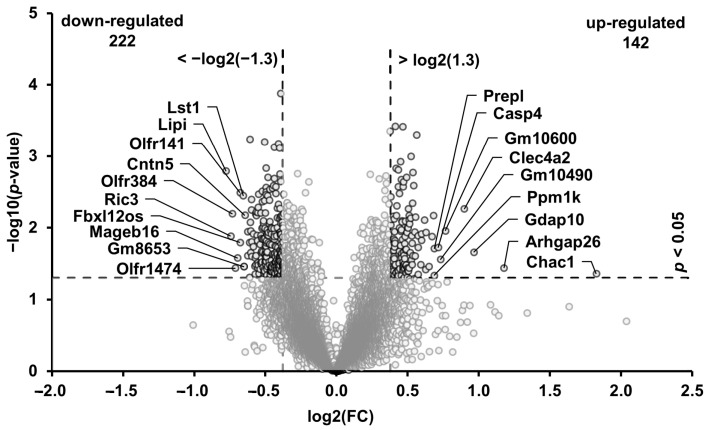
Volcano plot of differentially regulated transcripts in the *M. gastrocnemius* of adult male C57BL/6J mice. Mice were fed a diet containing either 0.3% (P_0.3_) or 1.2% (P_1.2_) phosphorus for 6 weeks. Horizontal (*p* < 0.05) and vertical (fold change (FC): >log2(1.3) or <−log2(−1.3)) dashed lines indicate double filtering criteria (*n* = 6 mice/group). The transcripts in the upper left and the upper right corners represent the down-regulated and up-regulated transcripts, respectively. The ten most up- or down-regulated transcripts are labeled.

A microarray analysis was performed to identify differentially expressed genes (DEGs) between the P_0.3_ and P_1.2_ groups in the *M. gastrocnemius* tissue. Of the total 22,206 transcripts analyzed, 222 were down-regulated and 142 were up-regulated in the P_1.2_ group compared to the P_0.3_ group, according to the two-filter criteria (FC > 1.3 or <−1.3, *p* < 0.05) (Figure 3). Among the up-regulated transcripts, two showed a FC > 2.0 (*Chac1* and *Arhgap26*), while none of the down-regulated transcripts showed a FC < −2.0. The 10 most up-regulated transcripts, in decreasing order of their FCs (in parentheses), were as follows: *Chac1* (3.54), *Arhgap26* (2.26), *Gdap10* (1.95), *Clec4a2* (1.86), *Gm10600* (1.70), *Gm10490* (1.66), *Casp4* (1.64), *Prepl* (1.61), and *Ppm1k* (1.61). The 10 transcripts that were most down-regulated, in ascending order of their FCs (in parentheses), were as follows: *Lipi* (−1.71), *Ric3* (−1.67), *Olfr384* (−1.66), *Olfr1474* (−1.64), *Mageb16* (−1.62), *Fbxl12os* (−1.60), *Olfr141* (−1.60), *Lst1* (−1.58), *Gm8653* (−1.57), and *Olfr872* (−1.57). The FCs and *p*-values of all the differentially expressed transcripts between groups P_0.3_ and P_1.2_ are listed in the Supplementary Materials (Table S4).

### 3.6. Technical Validation of the Microarray Data

The microarray data of 12 transcripts that were differentially expressed between the two groups were validated using qPCR. For 10 of the validated transcripts, the direction of the effect (positive or negative FC) was the same between the microarray and qPCR, while the magnitude of the effect (value of FC) differed, to some extent, between the microarray and qPCR. Statistical analysis of the qPCR data revealed that one (*Slc2a12*) of the validated transcripts was significantly regulated, as shown in the Supplementary Materials (*p* < 0.05, Table S3).

### 3.7. Biological Processes and Pathways Affected by the High-Phosphorus Diet

**Figure 4:**
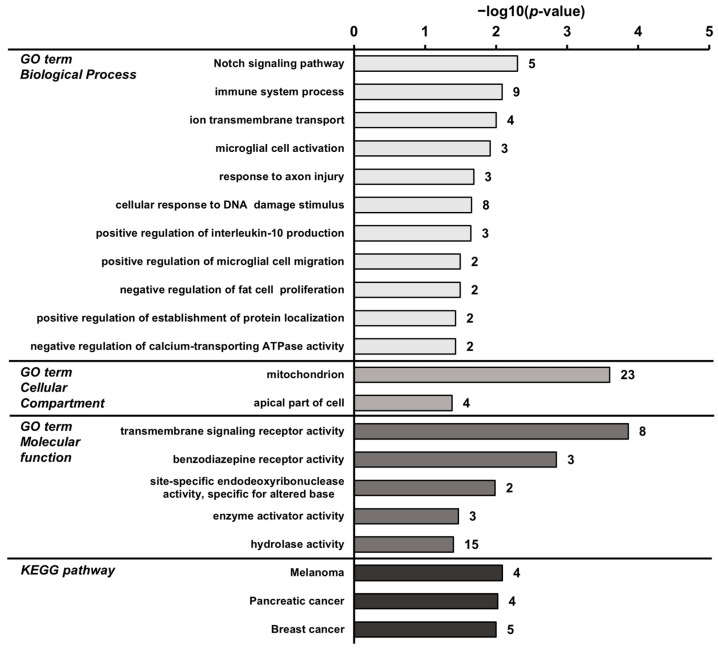
Enriched gene ontology (GO) terms by “biological process”, “cellular compartment”, “molecular function”, and KEGG pathways assigned to the transcripts that were up-regulated in the *M. gastrocnemius* by a high-phosphorus diet (P_1.2_) compared with an adequate-phosphorus diet (P_0.3_). Adult male C57BL/6J mice were fed a diet containing either 0.3% (P_0.3_) or 1.2% (P_1.2_) phosphorus for 6 weeks. GO terms and KEGG pathways are sorted using their enrichment *p*-values (EASE score) (top: lowest *p*-value; bottom: highest *p*-value). Only GO terms and KEGG pathways with *p* < 0.05 are shown. The number of linked up-regulated genes is indicated next to the bars. *n* = 6 mice/group.

**Figure 5:**
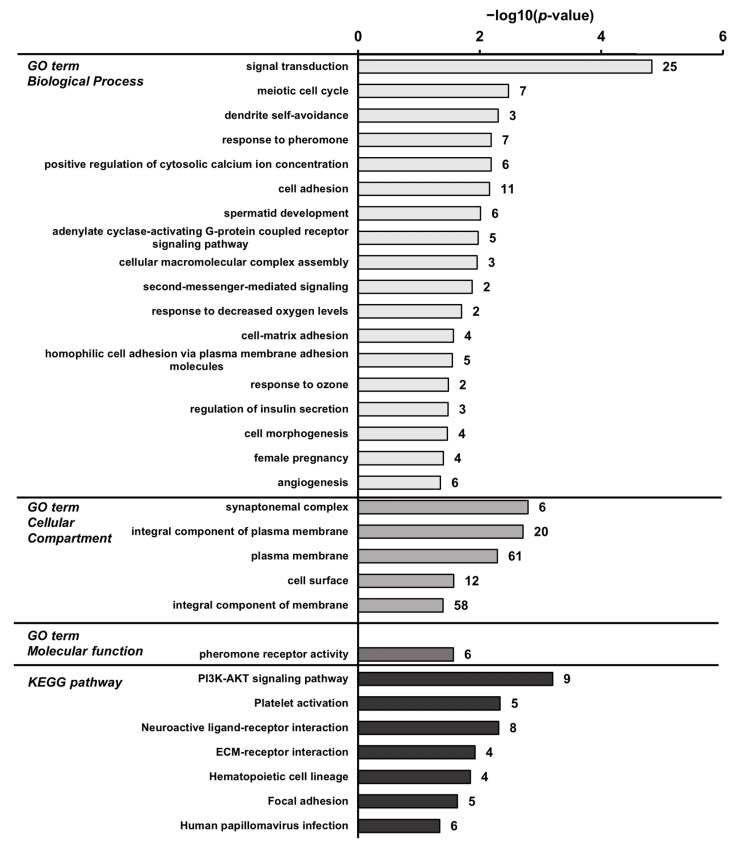
Enriched gene ontology (GO) terms by “biological process”, “cellular compartment”, “molecular function”, and KEGG pathways assigned to the transcripts that were down-regulated in the *M. gastrocnemius* by a high-phosphorus diet (P_1.2_) compared with an adequate-phosphorus diet (P_0.3_). Adult male C57BL/6J mice were fed a diet containing either 0.3% (P_0.3_) or 1.2% (P_1.2_) phosphorus for 6 weeks. GO terms and KEGG pathways are sorted using their enrichment *p*-values (EASE score) (top: lowest *p*-value; bottom: highest *p*-value). Only GO terms and KEGG pathways with *p* < 0.05 are shown. The number of linked down-regulated genes is indicated next to the bars. *n* = 6 mice/group.

To identify the biological processes and pathways that are associated with transcripts regulated by the high-phosphorus diet, GSEA was performed using the GO terms and Kyoto Encyclopedia of Genes and Genomes (KEGG) pathways. The GSEA results of the up-regulated transcripts in the P_1.2_ group compared to the P_0.3_ group identified 11 significant “biological process” terms that were influenced by the high-phosphorus diet. The most pronounced association was identified with the “biological process” term “Notch signaling pathway” (*p* = 0.005, Figure 4). The transcripts that were up-regulated in the P_1.2_ group compared to the P_0.3_ group were found to be predominantly enriched in the cancer-related KEGG pathways (Figure 4). More linked “biological process” terms (18 terms) were identified in the down-regulated transcripts (Figure 5). Signal transduction was the “biological process” to which the highest number of transcripts were assigned (*p* < 0.001, Figure 5). The most strongly linked KEGG pathway was the PI3K-AKT pathway (*p* < 0.001, Figure 5).

### 3.8. Effect of a High-Phosphorus Diet on Relative Muscle mRNA Abundance

**Figure 6:**
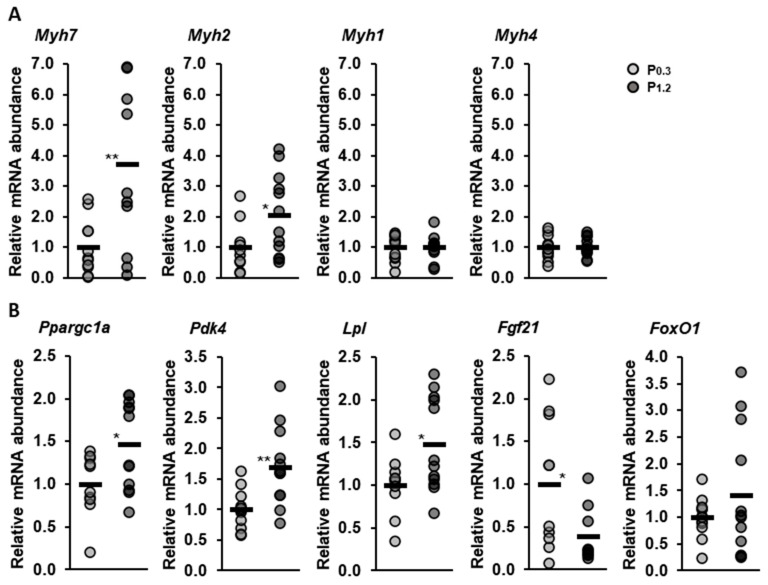
Normalized relative mRNA abundances of genes encoding myosin isoforms of muscle fiber types (**A**) and regulation of energy metabolism (**B**) in the *M. gastrocnemius* of adult male C57BL/6J mice determined using qPCR. Mice were fed a diet containing either 0.3% (P_0.3_) or 1.2% (P_1.2_) phosphorus for 6 weeks. Data are provided as single (circle) and mean values (horizontal line) (*n* = 12 mice/group) expressed as folds of relative mRNA abundance of the P_0.3_ group. * (*p* < 0.05) and ** (*p* < 0.01) next to the horizontal line indicate statistically significant differences. Abbreviations: *Myh7*, myosin heavy chain 7 encoding for myosin heavy chain (MHC)-I; *Myh2*, myosin heavy chain 2 encoding for MHC-IIa; *Myh1*, myosin heavy chain 1 encoding for MHC-IIx; *Myh4*, myosin heavy chain 4 encoding for MHC-IIb; *Ppargc1a*, peroxisome proliferative-activated receptor, gamma, coactivator 1 alpha; *Pdk4*, pyruvate dehydrogenase kinase, isoenzyme 4; *Lpl*, lipoprotein lipase; *Fgf21*, fibroblast growth factor 21; and *Foxo1*, forkhead box O1.

To evaluate the effect of a high-phosphorus diet on the muscle fiber composition, the gene transcriptions of the four predominant myosin isoforms that are typically used for the isoform typing of muscle fibers, namely MHC-IIx, MHC-IIa, MHC-IIb, and MHC-I, encoded by the *Myh1*, *Myh2*, *Myh4*, and *Myh7* genes, respectively, in the *M. gastrocnemius* were measured using qPCR. The mRNA abundances of *Myh7* and *Myh2* were significantly increased in the P_1.2_ group compared to the P_0.3_ group (*Myh7*, *p* = 0.006; *Myh2*, *p* = 0.039, Figure 6A), while no significant differences were observed in the mRNA abundances of *Myh1* and *Myh4* (*Myh1*, *p* = 0.983; *Myh4*, *p* = 0.982, Figure 6A).

Since PGC-1α is an important regulator of muscle fiber type composition, we also analyzed its mRNA abundance (*Ppargc1a*). The P_1.2_ group exhibited a higher mRNA abundance of *Ppargc1a* than the P_0.3_ group (*p* = 0.028, Figure 6B). Furthermore, the mRNA abundances of *Pdk4* and *Lpl* were higher in the P_1.2_ group than in the P_0.3_ group (*Pdk4*, *p* = 0.002; *Lpl*, *p* = 0.028, Figure 6B). The mRNA abundance of *Fgf21* was lower in the P_1.2_ group than in the P_0.3_ group (*p* = 0.044, Figure 6B). The mRNA abundance of *Foxo1* was not significantly different between the two groups (*p* = 0.262, Figure 6B).

### 3.9. Effect of a High-Phosphorus Diet on Relative Protein Levels in the Muscle

**Figure 7:**
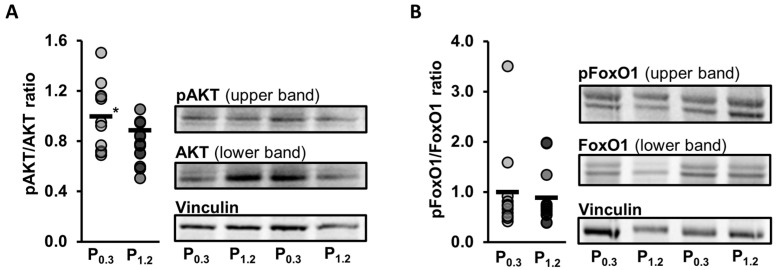
Normalized relative protein level of phosphorylated protein kinase B (pAKT) in relation to relative AKT level (**A**) and level of phosphorylated forkhead box O 1 (FoxO1) in relation to the level of FoxO1 (**B**), and representative images of the Western blot analysis in the *M. gastrocnemius* of adult male C57BL/6J mice. Mice were fed a diet containing either 0.3% (P_0.3_) or 1.2% (P_1.2_) phosphorus for 6 weeks. Data are provided as single (circle) and mean values (horizontal line) (*n* = 12 mice/group) expressed as fold ratios of the P_0.3_ group. * (*p* < 0.05) next to the horizontal line indicates statistically significant differences.

As the gene array analysis indicated an effect of the dietary phosphorus concentrations on the PI3K-AKT pathway, which aligns with previous research showing an influence of a high-phosphorus diet on this pathway [52], the protein levels of AKT and phosphorylated AKT (pAKT) were examined. While the high-phosphorus diet did not significantly affect the levels of AKT or pAKT (*p* > 0.05, Table 6), the ratio of pAKT to AKT was found to be significantly reduced by the high-phosphorus diet in relation to the adequate-phosphorus diet (*p* = 0.023, Figure 7A).

Furthermore, the transcriptome analysis showed a regulation of the Notch signaling pathway, which has been partly associated with muscle metabolism through the involvement of FoxO1. Therefore, the levels of FoxO1 and pFoxO1 were additionally analyzed. No significant effect of the high-phosphorus diet in relation to the adequate-phosphorus diet were observed on the protein levels of FoxO1 or pFoxO1 (*p* > 0.05, Table 6). In addition, the ratio of pFoxO1 to FoxO1 was also unaffected by the high-phosphorus diet in relation to the adequate-phosphorus diet (*p* = 0.723, Figure 7B).

## 4. Discussion

The present study tested the hypothesis that a high-phosphorus diet influences the lipid composition and gene transcription in the skeletal muscle of adult mice in relation to an adequate-phosphorus diet. In agreement with other studies [18,52,53], the body and liver weights were significantly reduced in the high phosphorus group in comparison to the adequate phosphorus group. Since the energy content of the two diets did not significantly differ and the energy and lipid digestibility were not influenced by the high-phosphorus diet, the body weight reduction in these mice was most likely due to the described effects on the lipid metabolism, like the suppressed transcription of lipogenic genes and the increased transcription of genes related to fatty acid β-oxidation [16], which were not further analyzed in this study. A strong reduction in the amount of visceral adipose tissue in the high phosphorus group was observed during sampling. Phosphorus digestibility was higher in the P_1.2_ group than in the P_0.3_ group, which could be due to the differences in the diet composition, as the P_0.3_ group received potassium bicarbonate as an additional source of potassium to balance the potassium content in the experimental diets. To ensure that the high-phosphorus diet did not result in a strong imbalance of the calcium and phosphorus homeostasis, plasma concentrations of calcium and inorganic phosphate were measured. The high-phosphorus diet had no effect on the plasma inorganic phosphate concentration but caused a reduction in the plasma calcium concentration in relation to the adequate-phosphorus diet. However, this reduction most likely had no physiological relevance, as the plasma calcium concentrations in both groups were within the normal range for adult mice [54], and the calcium digestibility was not influenced by the phosphorus concentration in the diet.

The weights of the *M. soleus*, *M. gastrocnemius*, and *M. rectus femoris* were not affected by the high-phosphorus diet. This is in agreement with Abuduli et al. [18], who reported that a high-phosphorus diet did not influence the muscle weight.

Since profound effects of the high phosphorus intake on lipid metabolism in the liver [16] and white and brown adipose tissue [18] have been described, it was of interest to investigate whether the high-phosphorus diet also influences the lipid concentrations of the skeletal muscle. Therefore, a lipidomic analysis of the *M. gastrocnemius* was carried out in the present study. The lipidome of the skeletal muscle was moderately altered by the high-phosphorus diet in relation to the adequate-phosphorus diet. In accordance with the literature, the predominant lipid classes in the muscle were TG, PC, and PE [31,55]. Within the 17 lipid classes that were analyzed, the concentrations of PE, PG, and LPC were significantly reduced in the high phosphorus group compared to the adequate phosphorus group. PE, PG, and LPC are all types of phospholipids. Phospholipids are major components of biological membranes. LPC is produced when PC is hydrolyzed using phospholipases and is involved in various signaling pathways. Analyzing the lipids at the level of individual species revealed that approximately 16% of the examined lipid species were significantly decreased due to the high-phosphorus diet. More than half of the regulated species were minor lipids. The PCA analysis did not show a separation of the two treatment groups, indicating, overall, only minor effects of the high-phosphorus diet on the individual lipid species.

Despite the minor changes in the lipidome and the unchanged proportions of the lipid classes, an overall reduction in the levels of certain lipids, particularly phospholipids, was observed. This finding is consistent with the effects of a high-phosphorus diet on liver lipid metabolism [16]. Chun et al. [16] observed an association between the liver transcriptome with the GO term “phospholipid metabolic process” when rats were fed a high-phosphorus diet; although, they did not analyze the phospholipid composition in the liver. However, in the present study, no such association was identified in the transcriptome of the muscle. Additionally, the concentration of total phospholipids in the muscle was unaffected by the high-phosphorus diet. Although little is known about the role of phospholipids in muscle metabolism, it has been shown that changes in the phospholipids in the mouse muscle can be linked to changes in other metabolic pathways, such as endoplasmic reticulum (ER) stress, mitochondrial dysfunction, and impaired cell homeostasis [56]. Considering that phospholipids are synthesized in the ER and that high phosphorus treatments have been shown to induce ER stress in vitro [57], it is possible that ER stress might also contribute towards the reduction in certain phospholipids in the muscle. Transcriptome analysis of human myotubes treated with LPC revealed influences on the peroxisome proliferator-activated receptor (PPAR) target transcripts, including ANGPTL4, PDK4, PLIN2, and CPT1A [58].

To investigate the effect of the high phosphorus intake on the muscle transcriptome, and to evaluate whether the changes in the muscle’s lipidome are also reflected in changes in the gene transcription, a gene array analysis was carried out. Consistent with the lipidome data, the influences of a high phosphorus intake on the muscle transcriptome were limited. Only a relatively small number of genes was regulated by the high-phosphorus diet, with the number of genes that were down-regulated being greater than the number of genes that were up-regulated, in relation to the adequate-phosphorus diet. The strongest connection of transcripts reduced by the high-phosphorus diet detected through GSEA was with “signal transduction”. Most of the genes connected were G-protein coupled receptors; however, the *Lep* gene was also down-regulated, which encodes for the hormone leptin, which is involved in the regulation of appetite and energy expenditure. Regarding the connection with the “KEGG pathway”, the most pronounced link of the down-regulated genes was identified with the PI3K-AKT-signaling pathway. Phosphorylation plays a crucial role in the activation of the AKT protein. Interestingly, Western blot analysis revealed a decrease in the ratio of phosphorylated AKT (pAKT) to unphosphorylated AKT protein, indicating a lower activation of the AKT protein. A similar observation was also shown in the heart muscle of mice fed a high-phosphorus diet and was accompanied with a lower heart weight [52]. The PI3K-AKT signaling pathway is a crucial pathway involved in numerous cellular processes, including cell growth, survival, and metabolism. In muscle tissue, this pathway plays a vital role in regulating muscle growth and the uptake of glucose. When insulin binds to its receptor on the muscle cell surface, it activates the PI3K-AKT pathway. This results in the stimulation of downstream signaling molecules, such as mTOR and GSK-3 (glycogen synthase kinase 3), which promote the synthesis of proteins and inhibit their degradation, leading to muscle growth [59]. Additionally, AKT activation also stimulates the translocation of glucose transporter type 4 (GLUT4) to the muscle cell membrane, promoting glucose uptake and metabolism in muscle cells [60]. An inhibition of this pathway could lead to reduced muscle growth and a reduced glucose uptake in the muscle. The present study did not indicate a reduction in muscle growth as the weights of the skeletal and heart muscles were not influenced by the high-phosphorus diet. However, it is important to note that the mice used in this study were adults and already fully grown. Chung et al. [61] reported that a high phosphorus treatment could promote muscle atrophy. In vitro analysis of myotubes exposed to high phosphorus concentrations resulted in a reduction in the size of the myotubes, increased generation of reactive oxygen species, decreased protein synthesis, and accelerated protein degradation. However, in vivo experiments involving feeding a high-phosphorus diet to 10-week-old sham-operated and chronic kidney disease (CKD) mice for 20 weeks did not show any noticeable effects on the body weight, *M. gastrocnemius* weight, or grip strength. Furthermore, feeding a high-phosphorus diet increased the nuclear levels of phosphorylated Nrf2 (a transcription factor involved in the cellular stress response) in the *M. gastrocnemius* from both the sham-operated and CKD mice, and increased nuclear levels of phosphorylated p62 (a protein associated with autophagy). On the other hand, a reduction in glucose uptake in the muscle induced by PI3K-AKT signaling could lead to an increase in β-oxidation. This is further supported with the up-regulation of genes that are associated with mitochondria, as the GSEA has indicated. The GO category “cellular compartment” analysis revealed that 23 up-regulated genes were associated with mitochondria, which play a fundamental role for ATP provision in the muscle and are the site for β-oxidation. These included genes, such as mitochondrial trans-2-enoyl-CoA reductase (*Mecr*), the protein encoded by *Mecr*, which is an oxidoreductase that catalyzes the final step of mitochondrial fatty acid synthesis, or acyl-CoA synthetase long chain family member 5 (*Acsl5*), which converts free long-chain fatty acids into fatty acyl-CoA esters, thus play a key role in lipid biosynthesis and fatty acid degradation. The upregulation of these genes could indicate an increased demand for β-oxidation and a higher utilization of fatty acids as an energy source in the muscle tissue.

In relation to the GO category “biological process”, the analysis revealed a notable association of the up-regulated transcripts with the Notch signaling pathway. The Notch signaling pathway is a highly conserved cell signaling system that plays a crucial role in various developmental processes, including cell proliferation, differentiation, and apoptosis. Studies have highlighted the role of the Notch signaling pathway in muscle metabolism. In the skeletal muscle, Notch signaling has been shown to regulate myogenic differentiation, muscle growth, and muscle regeneration [62]. Activation of the Notch pathway in muscle stem cells promotes their proliferation and differentiation into myoblasts, which subsequently fuse to form mature myotubes [63]. In addition to its well-established role in myogenesis, recent studies have suggested a potential involvement of Notch signaling in the regulation of muscle metabolism. Interestingly, the interaction between Notch and FoxO1, a transcription factor, appears to be essential in this context. However, in the current study, no influence of the high-phosphorus diet was observed on the *Foxo1* gene transcription or protein levels, nor on its inactivation via phosphorylation, indicating tat the observed regulation of the Notch signaling pathway and its potential impact on muscle metabolism may involve alternative mechanisms or signaling pathways that were not directly assessed in this study. In the skeletal muscle, the gene array showed an upregulation of the following genes which are connected to the Notch signaling pathway: *Cdh6*, *Ptp4a3*, *Jag1*, *Dtx3l*, and *Chac1*. *Cdh6* encodes for cadherin 6, a transmembrane protein involved in cell adhesion and signaling. *Ptp4a3* encodes for protein tyrosine phosphatase type IVA member 3, which plays a role in the regulation of cell proliferation, migration, and survival. *Jag1* encodes for Jagged-1, a transmembrane protein that is a ligand for the Notch signaling pathway. *Dtx3l* encodes for Deltex E3 ubiquitin ligase 3-like protein, which is involved in the regulation of the Notch signaling pathway. *Chac1* encodes for cysteine-rich angiogenic inducer 61 (CYR61)/connective tissue growth factor (CTGF)/nephroblastoma overexpressed (NOV) protein-associated protein 1 (NOR1) C-terminal (CHC1) domain-containing protein 1, which is involved in the regulation of the cellular stress response. *Chac1* has been shown to be important across various biological processes, including the unfolded protein response, apoptosis, and inflammation.

Despite the relatively modest effects observed in the muscle lipidome and transcriptome as a result of a high-phosphorus diet, the data consistently indicate an impact on fatty acid metabolism in the muscles. Specifically, these findings suggest an increase in β-oxidation within the muscles. This interpretation is supported by the elevated gene transcription of *Ppargc1a*, encoding the transcriptional PGC-1α, *Pdk4,* and *Lpl*, which are all associated with the utilization of fatty acids in the muscle. The skeletal muscle is a heterogeneous tissue that exhibits a high level of metabolic flexibility and is composed of fibers with different morphological and functional properties [64,65]. Classification of muscle fibers can be based on certain characteristics, such as contractile speed, myosin heavy chain (MHC) expression, and metabolic capacity [65,66]. In the adult mammalian muscle, four main MHC isoforms can be distinguished: one slow isoform (MHC-I) and three fast isoforms (MHC-IIa, MHC-IIx, and MHC-IIb). However, human skeletal muscle does not contain MHC-IIb [65,67,68]. Type II fibers (“glycolytic fibers”) have a small number of mitochondria and mainly generate ATP mainly through glycolytic metabolism; in contrast, type I fibers (“oxidative fibres”) are rich in mitochondria and therefore mainly use oxidative phosphorylation [69,70]. Since muscle tissue has a remarkable capacity to undergo adaptive changes, the distribution of type I and II fibers can be influenced by various factors, such as exercise, mechanical unloading, obesity, or diabetes, resulting in changes in the functional and metabolic phenotypes of the muscle [71,72,73,74]. The results of the qPCR data show that the high-phosphorus diet led to a three- to four-fold increase in the gene transcription of *Myh7* (MHC-I) and an increase in the gene transcription of *Myh2* (MHC-IIa) in the *M. gastrocnemius* compared to the adequate-phosphorus diet. The gene transcriptions of *Myh1* (MHC-IIx) and *Myh4* (MHC-IIb) were not affected by the high-phosphorus diet compared to the adequate-phosphorus diet. In line with the qPCR data, the transcriptome analysis showed a higher, but not statistically significant, level of transcription of *Myh7* (FC = 1.24; *p* > 0.05) and *Myh2* (FC = 1.27; *p* > 0.05) in the high phosphorus group compared to the adequate phosphorus group. Also, comparable to the qPCR analysis, transcriptome analysis showed that the high-phosphorus diet did not affect the transcription of *Myh1* (FC = 1.03; *p* > 0.05) or *Myh4* (FC = −1.00; *p* > 0.05). Therefore, from the results of the qPCR and the transcriptional analysis, it can be inferred that a high-phosphorus diet could lead to a higher abundance of type I (oxidative and slow twitch) fibers. In association with muscle fiber transformation, qPCR indicated an increased transcription of the gene *Ppargc1a* in the high phosphorus group. In addition, it was further observed that the gene transcription of *Ppargc1a* was significantly related to the transcription of *Myh7* (Pearson correlation coefficient (r) = 0.756; *p* < 0.001) and *Myh2* (r = 0.608; *p* < 0.01). PGC-1α functions as an essential transcriptional regulator, which is involved in the contractile and metabolic muscle fiber transformation towards the oxidative fiber type [75,76,77]. Lin et al. [77] showed that PGC-1α is manly expressed in the oxidative type I fibers. In a more extensive gain-of-function mouse experiment designed to investigate the role of PGC-1α in the muscle fiber specification, the authors observed that the overexpression of PGC-1α in the muscle leads to the transformation of the muscle fibers.

A shift from type II to type I fibers would indicate that the muscle adapts to highly oxidative aerobic metabolism for energy production, which stands in contrast to a previous study [27], in which a high-phosphorus diet compared to an adequate-phosphorus diet led to an impaired exercise tolerance and fatty acid metabolism. In this previous study, the gene expression was measured in the muscle tissue, which was selected immediately after physical exercise. A switch to the type I muscle fiber would actually improve exercise endurance and fatty acid utilization, as type I muscle fibers are characterized by their high oxidative capacity, high resistance to fatigue, and low contractile speed [78]. These fibers contain a high concentration of mitochondria and myoglobin, which allows for the efficient use of oxygen in energy production [79]. Additionally, type I fibers have a smaller cross-sectional area and a higher capillary density compared to type II fibers, which further enhances their aerobic capacity [80]. These fibers express high levels of genes involved in oxidative metabolism, including mitochondrial enzymes and respiratory chain proteins [81], enabling them to utilize fatty acids, particularly long-chain fatty acids, as an energy source. Moreover, they exhibit lower activity levels of glycolytic enzymes and fast contraction, such as myosin heavy chain II isoforms [82]. This is also consistent with the increased gene transcription of *Lpl*. LPL is an important enzyme that plays a critical role in lipid metabolism in muscle tissue. LPL is primarily expressed in the skeletal muscle and adipose tissue, where it catalyzes the hydrolysis of triacylglycerol-rich lipoproteins into free fatty acids for energy utilization. In muscle tissue, LPL activity is regulated by several factors, including insulin, exercise, and diet. Additionally, the gene transcription of *Fgf21* was reduced in the high phosphorus group compared to the adequate phosphorus group, which may also play a role in regulating muscle metabolism, as it has been shown to increase glucose uptake and utilization in muscle tissue [83]; a reduction would be in accordance with a switch to the oxidative fiber type I.

The conflicting data regarding the impact of a high phosphorus intake on muscle metabolism and its metabolic energy utilization emphasizes the necessity for additional research in this area. While some studies have suggested effects on fatty acid metabolism and β-oxidation, the overall understanding of the relationship between a high-phosphorus diet and muscle metabolism remains limited.

## 5. Conclusions

Overall, this study shows that a high-phosphorus diet has moderate effects on the skeletal muscle lipidome and transcriptome. The lipidome analysis showed a reduction in the concentration of specific lipids, especially of PE, PG, and LPC, but no significant changes in the lipid composition. The transcriptome analysis showed limited effects of the high-phosphorus diet on gene transcription; the gene ontology analysis revealed an enrichment of genes associated with mitochondria, suggesting a possible role of a high phosphorus intake in mitochondrial function and energy homeostasis. While utilizing the unadjusted raw *p*-values for identifying significantly altered lipid species and gene transcripts represents a limitation of this study, the data itself demonstrate remarkable consistency and highlights an overall impact of a high-phosphorus diet on lipid metabolism within the muscle. The results of the lipidome and transcriptome analyses suggest that there is a relationship between high phosphorus intake and increased β-oxidation of lipids in the muscle. Additionally, the increased transcription of *Myh7*, which encodes MHC-I, a type I oxidative fiber-specific protein in muscle, indicates that muscle fiber switching may occur due to a high phosphorus intake. Due to technical limitations in sampling, the detection at the protein level could not be conducted in this study. Therefore, further investigations are needed to analyze this aspect in future studies and to elucidate the mechanisms underlying these findings, as well as to evaluate the potential health implications of a high-phosphorus diet on skeletal muscle metabolism. Overall, although the influences were limited, the study showed that a high-phosphorus diet could affect skeletal muscle lipid metabolism and gene transcription.

## Figures and Tables

**Table 1:** Composition of the adequate- and the high-phosphorus diets.

Component (g/kg)	Adequate-Phosphorus Diet (0.30% Phosphorus)	High-Phosphorus Diet (1.20% Phosphorus)
Cornstarch	524.05	513.61
Casein ^2^	200	200
Sucrose	100	100
Soybean oil	50	50
Cellulose	50	50
Mineral mix ^1^	25	25
KH_2_PO_4_ ^2,3^	6.86	46.39
KHCO_3_ ^4^	29.09	-
Vitamin mix ^5^	10	10
Titanium dioxide	5	5

^1^ The mineral mix provided the following per kg diet: calcium, 5 g; chloride, 1.57 g; sodium, 1.02 g; magnesium, 0.51 g; iron, 35 mg; zinc, 30 mg; manganese, 10 mg; copper, 6 mg; chromium, 1 mg; fluoride, 1 mg; iodate, 0.2 mg; molybdate, 0.15 mg; selenium; 0.15 mg; and lithium, 0.10 mg. ^2^ Casein provided 1.44 g phosphorus per kg diet. ^3^ KH_2_PO_4_ provided the following per kg diet: 1.561 g of phosphorus, 1.97 g of potassium in the 0.30% diet and 10.56 g of phosphorus, and 13.33 g of potassium in the 1.20% diet. ^4^ KHCO_3_ provided 11.36 g of potassium per kg of the 0.30% diet. ^5^ The vitamin mix provided the following per kg diet: choline, 1 g; all-rac-α tocopherol acetate, 75 mg; nicotinic acid, 30 mg; pantothenic acid, 15 mg; riboflavin, 6 mg; pyridoxine HCl, 6 mg; thiamine HCl, 5 mg; folic acid, 2 mg; all-trans-retinol, 1.38 mg; menadione sodium bisulfate, 0.75 mg; biotin, 0.2 mg; cholecalciferol, 0.025 mg; and cyanocobalamin, 0.025 mg.

**Table 2:** Body and organ weights, as well as feed and energy intake, of adult male C57BL/6J mice. Mice were fed diets with either 0.3% (P_0.3_) or 1.2% (P_1.2_) phosphorus for 6 weeks.

	P_0.3_	P_1.2_	*p*-Value
Body weight, g			
Initial	29.8 ± 1.8	29.8 ± 2.0	0.960
Week 1	30.1 ± 1.6	29.6 ± 2.3	0.563
Week 2	30.7 ± 1.6	30.4 ± 2.3	0.679
Week 3	31.6 ± 1.4	30.7 ± 2.0	0.235
Week 4	32.3 ± 1.6	31.2 ± 2.1	0.162
Week 5	33.0 ± 1.6	31.4 ± 1.8	0.033
Week 6	34.0 ± 1.6	31.7 ± 1.8	0.003
Feed and energy intake			
Daily feed intake, g	3.77 ± 0.44	3.87 ± 0.58	0.759
Daily energy intake, KJ	62.6 ± 7.3	63.7 ± 9.6	0.821
Tibia length and organ weights			
*Tibia* length right, cm	1.78 ± 0.08	1.79 ± 0.02	0.730
*Tibia* length left, cm	1.80 ± 0.03	1.79 ± 0.02	0.294
Heart, mg	151 ± 12	159 ± 25	0.324
Kidney right, mg	177 ± 11	194 ± 32	0.099
Liver, g	1.54 ± 0.14	1.16 ± 0.24	<0.001
Liver to body weight ratio, mg/g	4.53 ± 0.40	3.65 ± 0.71	0.001
*M. soleus* right, mg	9.75 ± 1.19	9.61 ± 1.48	0.799
*M. gastrocnemius* right, mg	170 ± 15	171 ± 14	0.871
*M. rectus femoris* right, mg	218 ± 47	208 ± 53	0.627

Data are means and standard deviations (*n* = 12 mice/group and *n* = 6 cages/group for feed and energy intake, respectively).

**Table 3:** Apparent total tract digestibility (ATTD) of nutrients in adult male C57BL/6J mice. Mice were fed a diet containing either 0.3% (P_0.3_) or 1.2% (P_1.2_) phosphorus for 6 weeks.

	P_0.3_	P_1.2_	*p*-Value
ATTD, %			
Phosphorus	40.4 ± 3.9	73.3 ± 1.0	0.002
Calcium	5.09 ± 3.03	5.13 ± 2.17	0.222
Crude ash	54.5 ± 1.0	63.0 ± 0.8	0.001
Crude lipids	97.7 ± 0.5	96.5 ± 0.4	0.001
Gross energy	91.2 ± 0.4	90.6 ± 0.4	0.024

Data are means and standard deviations (*n* = 6 cages/group).

**Table 4:** Plasma concentrations of calcium and inorganic phosphate in adult male C57BL/6J mice. Mice were fed a diet containing either 0.3% (P_0.3_) or 1.2% (P_1.2_) phosphorus for 6 weeks.

	P_0.3_	P_1.2_	*p*-Value
Plasma concentration, mmol/L			
Calcium	2.42 ± 0.10	2.27 ± 0.13	0.004
Inorganic phosphate	2.16 ± 0.15	2.26 ± 0.19	0.127

Data are means and standard deviations (*n* = 12 mice/group).

**Table 5:** Concentrations of lipid classes in the *M. gastrocnemius* of adult male C57BL/6J mice. Mice were fed a diet containing either 0.3% (P_0.3_) or 1.2% (P_1.2_) phosphorus for 6 weeks.

	P_0.3_	P_1.2_	*p*-Value
Major lipid classes, nmol/mg wet weight			
Triacylglycerol	7.61 ± 8.83	6.22 ± 5.66	0.987
Phosphatidylcholine (PC)	6.76 ± 1.05	6.09 ± 0.72	0.092
Phosphatidylethanolamine (PE)	2.45 ± 0.64	1.93 ± 0.34	0.027
Phosphatidylinositol	1.69 ± 0.19	1.67 ± 0.14	0.747
Free cholesterol	1.37 ± 0.47	1.48 ± 0.39	0.427
Minor lipid classes, pmol/mg wet weight			
Phosphatidylserine	678 ± 103	669 ± 69	0.895
PE-based plasmalogens	676 ± 171	697 ± 126	0.625
Cardiolipin	438 ± 155	325 ± 118	0.060
Sphingomyelin	258 ± 62	271 ± 45	0.428
Diacylglycerol	164 ± 88	121 ± 30	0.058
Phosphatidylglycerol	94.9 ± 18.1	77.4 ± 11.8	0.011
PC ether	67.1 ± 44.3	81.5 ± 51.7	0.419
Hexosylceramide	61.0 ± 90.8	87.8 ± 106	0.584
Lysophosphatidylcholine	51.5 ± 12.8	38.4 ± 7.1	0.004
Ceramide	36.1 ± 5.7	35.1 ± 3.8	0.685
Lysophosphatidylethanolamine	27.3 ± 9.9	24.2 ± 7.6	0.422
Cholesteryl ester	5.60 ± 3.73	4.89 ± 8.61	0.119
Sums, nmol/mg wet weight			
Total	22.4 ± 9.7	19.8 ± 5.1	0.495
Phospholipids	13.2 ± 2.3	11.9 ± 1.2	0.090
Lipid composition, % of total lipids			
Phosphatidylcholine	33.4 ± 9.8	32.6 ± 8.5	0.898
Triacylglycerol	27.0 ± 21.5	27.0 ± 19.6	0.420
Phosphatidylethanolamine	12.1 ± 4.3	10.5 ± 3.7	0.534
Phosphatidylinositol	8.46 ± 2.56	8.88 ± 2.09	0.383
Free cholesterol	6.59 ± 2.03	7.97 ± 2.96	0.564
Phosphatidylserine	3.36 ± 1.01	3.58 ± 0.92	0.332
PE-based plasmalogens	3.27 ± 0.92	3.74 ± 1.14	0.136
Cardiolipin	2.17 ± 0.91	1.83 ± 0.96	0.661
Sphingomyelin	1.25 ± 0.34	1.45 ± 0.41	0.464
Diacylglycerol	0.734 ± 0.132	0.575 ± 0.238	0.095
Phosphatidylglycerol	0.461 ± 0.119	0.413 ± 0.110	0.357
PC ether	0.303 ± 0.112	0.451 ± 0.333	0.927
Lysophosphatidylcholine	0.247 ± 0.067	0.206 ± 0.063	0.266
Hexosylceramide	0.235 ± 0.231	0.498 ± 0.643	0.227
Ceramide	0.183 ± 0.063	0.187 ± 0.046	0.092
Lysophosphatidylethanolamine	0.129 ± 0.040	0.133 ± 0.059	0.829
Cholesteryl ester	0.0234 ± 0.0088	0.0209 ± 0.0305	0.243

Data are means and standard deviations (*n* = 12 mice/group).

**Table 6:** Normalized relative protein levels of phosphorylated (p) and unphosphorylated protein kinase B (AKT) and forkhead box O 1 (FoxO1) in the *M. gastrocnemius* of adult male C57BL/6J mice. Mice were fed a diet containing either 0.3% (P_0.3_) or 1.2% (P_1.2_) phosphorus for 6 weeks.

	P_0.3_	P_1.2_	*p*-Value
Relative protein level			
AKT	1.00 ± 0.31	1.20 ± 0.50	0.277
pAKT	1.00 ± 0.36	0.93 ± 0.42	0.655
FoxO1	1.00 ± 0.68	1.10 ± 0.87	0.945
pFoxO1	1.00 ± 0.39	0.98 ± 0.45	0.890

Protein levels were normalized to the reference protein vinculin. Data are means and standard deviations (*n* = 12 mice/group) as folds of the relative protein level of the P_0.3_ group.

## Data Availability

The microarray data have been deposited in MIAME compliant format in the NCBI’s Gene Expression Omnibus public repository (GEO accession no. GSE236311). The other datasets used and analyzed during the current study are available from the corresponding author on reasonable request.

## Supplementary Materials

The following supporting information can be downloaded at: https://www.mdpi.com/article/10.3390/nu15173734/s1, Table S1. Characteristics of gene-specific primers used for qPCR analysis in the *M. gastrocnemius* of adult male C57BL/6J mice. Mice were fed a diet containing either 0.3% (P_0.3_) or 1.2% (P_1.2_) phosphorus for 6 weeks. Table S2. Individual lipid species significantly (*p* < 0.05) affected in the *M. gastrocnemius* of adult male C57BL/6J mice fed a diet containing either 0.3% (P_0.3_) or 1.2% (P_1.2_) phosphorus for 6 weeks. Table S3. qPCR validation of the transcriptome data for selected differentially expressed transcripts (fold change (FC) > 1.3 or <−1.3, *p* < 0.05) in the *M. gastrocnemius* of adult male C57BL/6J mice. Mice were fed a diet containing either 0.3% (P_0.3_) or 1.2% (P_1.2_) phosphorus for 6 weeks. Table S4. Fold change (FC) and *p*-values of all differentially expressed transcripts (FC > 1.3 or <−1.3, *p* < 0.05) in the *M. gastrocnemius* of adult male C57BL/6J mice. Mice were fed a diet containing either 0.3% (P_0.3_) or 1.2% (P_1.2_) phosphorus for 6 weeks.
