# Supplementary material for: A High-Phosphorus Diet Moderately Alters the Lipidome and Transcriptome in the Skeletal Muscle of Adult Mice
Source: Nutrients. 2023 Aug 25;15(17):3734. doi: 10.3390/nu15173734 (PMC10489812; doi:10.3390/nu15173734)
Supplement: Supplementary file 1 [file nutrients-15-03734-s001.zip › nutrients-2530873-supplementary.pdf]

nutrients

MDPI

Supplementary

Table S1. Characteristics of gene-specific primers used for qPCR analysis in the *M. gastrocnemius* of adult male C57BL/6J mice. Mice were fed a diet containing either 0.3% ($P_{0.3}$) or 1.2% ($P_{1.2}$) phosphorus for 6 weeks.

Gene Symbol[1]	Name	Primer sequence	Accession	Annealing (°C)	Amplicon size (bp)
Reference genes					
Actb	actin, beta	AGTGTGACGTTGACATCCGT<br>AGCTCAGTAACAGTCCGCCTA	NM_007393.5	60	296
B2m	beta-2 microglobulin	CTCGGTGACCCTGGTCTTTC<br>CATGCTTAACTCTGCAGGCG	NM_009735.3	60	305
Ppia	peptidylprolyl isomerase A	GCGTCTCCTTCGAGCTGTTT<br>ATGCCTTCTTTCACCTTCCCA	NM_008907.2	59	352
Target genes					
Akt2	thymoma viral proto-oncogene 2	TCAAACTCCTCGGCAAGGGC<br>TCACCCCCGTTGGCATACTC	NM_007434.4	60	244
Chac1	ChaC, cation transport regulator 1	TCCATAGGGGCAGCGACAAG<br>AGTGCTGTGAGGGGTTGGTC	NM_026929.4	60	214
Fbxl12os	F-box and leucine-rich repeat protein 12, opposite strand	GGGTGGCACCGATTCCTCTT<br>CCTCGCTCTTCGCTCAGACT	NR_033729.1	62	118
Fgf21	fibroblast growth factor 21	CCTGCGTCTGCCTCAGAAGG<br>GAGTCAGGACGCATAGCTGGG	NM_020013.4	63	205
Foxo1	forkhead box O1	GAGAAGAGGCTCACCCTGTCG<br>TTGCCTCCCTCTGGATTGAGC	NM_019739.3	60	203
Gdap10	ganglioside-induced differentiation-associated-protein 10	GCCTGGCACGTAGAACAAAC<br>AGTTTGCTGCCCTCGTTACT	NR_045032.2	60	104
Lpl	lipoprotein lipase	TGAGGATGGCAAGCAACACA<br>TTGTTTGTCCAGTGTCAGCC	NM_008509.2	57	384
Lrp2bp	Lrp2 binding protein	GCAGCGAGAAGTTGCCCAAA<br>TCCTCAAACTGTGCCAAGGCT	NM_026278.3	60	238
Mageb16	MAGE family member B16	TGGTCTTTGGGCTGGATGTG<br>AATCTCCTCTTCAGTGGCACG	NM_028025.1	60	185
Myh1	myosin, heavy polypeptide 1	GGAAGCCCGCAATGCAGAAGAG<br>TGTTTGCGCAGACCCTTGATAGC	NM_030679.2	62	291
Myh2	myosin, heavy polypeptide 2	GGACGACTGCAGACCGAATCC<br>AGCCTTAGACTCCTGCTCCTCC	NM_001039545.2	64	234
Myh4	myosin, heavy polypeptide 4	AGCTGGAGTTGAACCAGGTGA<br>CATCATTCCTGCTGCGGATCT	NM_010855.3	60	147
Myh7	myosin, heavy polypeptide 7	CAGGAGAAGAAGGTGCGCATGG<br>GCTTGCTCATCCTCAATCCTGGC	NM_080728.3	64	179

Olfr1474	olfactory receptor 1474	ACTTGGTGCTACACTTGTGGG<br>TGTAGCCAAGACGAGCAGTG	AY318693.1	60	177
Olfr384	olfactory receptor 384	CCCATCTCCACACACCCATGT<br>GGAGATGGATGGGTCCTGGC	AY317484.1	60	122
Pdk4	pyruvate dehydrogenase kinase, isoenzyme 4	CCTGCCTGACCGCTTAGTGAA<br>GGCTTTCTGGTCTTCTGGGCT	AF239176.1	58	115
Ppargc1a	peroxisome proliferative activated receptor, gamma, coactivator 1 alpha	CTGGGTGGATTGAAGTGGTG<br>TCAGTGCATCAAATGAGGGC	NR_027710.2	60	184
Ric3	RIC3 acetylcholine receptor chaperone	TGGAAACGCCCACAGGAAGAT<br>ACCCTGGTGATTTCTCGGAGC	NM_001038624.1	60	186
Sfxn2	sideroflexin 2	TGGCAGTGGGTGAACCAGTC<br>GGCCACCGCAGTAGTAGTAGC	AF325261.1	60	123
Slc2a12	solute carrier family 2 (facilitated glucose transporter), member 12	CACTCCCTACAGCAAGGGCT<br>CACTAAGCACCAACCAAGGCA	NM_178934.4	60	236
Tmem45b	transmembrane protein 45b	CTCTGAGGCTCGGAAGAGGC<br>GCTCTGCCAGGATCCCAATGA	NM_144936.1	62	233

[1] Eurofins Genomics (Ebersberg, Deutschland)

Table S2. Individual lipid species significantly ($p < 0.05$) affected in the *M. gastrocnemius* of adult male C57BL/6J mice fed a diet containing either 0.3% ($P_{0.3}$) or 1.2% ($P_{1.2}$) phosphorus for 6 weeks.

**Individual lipid species**	$P_{0.3}$	$P_{1.2}$	***P*-value**
Cholesterol ester (CE)			
CE 22:6	0.372 ± 0.188	0.204 ± 0.105	0.013
CE 20:4	2.16 ± 1.10	1.07 ± 0.55	0.006
Ceraminde (Cer)			
Cer 18:1;O2/22:0	2.08 ± 0.71	1.52 ± 0.40	0.026
Cardiolipin (CL)			
CL 70:7	14.8 ± 5.8	10.1 ± 3.7	0.027
CL 72:7	54.5 ± 25.2	35.6 ± 18.3	0.047
CL 72:8	55.6 ± 29.8	33.5 ± 21.1	0.047
CL 80:10	2.89 ± 2.42	1.03 ± 1.59	0.036
CL 72:10	6.00 ± 2.29	4.17 ± 2.02	0.049
CL 76:12	22.3 ± 9.6	15.0 ± 7.5	0.049
CL 70:6	17.9 ± 5.5	13.7 ± 3.6	0.032
CL 72:9	10.3 ± 3.2	7.89 ± 2.44	0.045
Diacylglycerol (DG)			
DG 32:0	21.0 ± 5.39	15.6 ± 4.7	0.018
DG 38:5	5.23 ± 1.67	3.14 ± 1.36	0.004
Lysophosphatidylcholine (LPC)			
LPC 18:1	4.32 ± 1.48	3.04 ± 0.62	0.011

LPC 18:3	0.167	±	0.055	0.117	±	0.031	0.013
LPC 20:4	2.71	±	1.18	1.88	±	0.43	0.031
LPC 22:6	4.38	±	2.51	2.70	±	1.08	0.045
LPC 16:1	0.947	±	0.366	0.654	±	0.085	0.013
LPC 18:2	5.04	±	1.82	3.62	±	1.30	0.039
LPC 16:0	18.9	±	3.9	14.9	±	3.0	0.010
LPC 18:0	10.6	±	1.9	7.82	±	1.29	0.000
LPC 20:0	0.284	±	0.090	0.191	±	0.063	0.008
LPC 20:3	0.488	±	0.141	0.281	±	0.110	0.001
Phosphatidylcholine (PC)							
PC 36:2	173	±	63	129	±	27	0.034
PC 32:1	276	±	45	232	±	39	0.017
PC 34:2	611	±	105	518	±	59	0.014
PC 36:3	158	±	23	134	±	28	0.037
PC ether (PC O)							
PC O-36:5	11.6	±	1.4	13.5	±	2.3	0.022
Phosphatidylethanolamine (PE)							
PE 34:2	12.4	±	2.7	9.68	±	1.12	0.004
PE 34:3	1.78	±	0.43	1.49	±	0.20	0.044
PE 38:4	181	±	66	126	±	26	0.013
PE 40:6	1250	±	448	903	±	258	0.028
PE 42:7	11.2	±	3.55	8.07	±	2.35	0.020
PE 36:3	8.03	±	2.50	6.03	±	0.80	0.015
PE 36:5	1.98	±	0.39	1.63	±	0.13	0.007
PE 32:1	2.70	±	0.38	2.24	±	0.20	0.001
PE 36:4	21.4	±	3.2	17.7	±	2.0	0.003
PE 38:3	9.00	±	2.37	7.00	±	1.71	0.027
PE 38:6	544	±	60	465	±	82	0.013
PE 42:6	5.26	±	1.88	3.71	±	0.99	0.019
PE based plasmalogens (PE P)							
PE P-16:0/18:2	5.25	±	0.97	4.57	±	0.42	0.036
PE P-16:0/20:5	1.09	±	0.34	0.705	±	0.226	0.003
PE P-18:0/20:5	0.710	±	0.228	0.479	±	0.163	0.009
PE P-18:1/20:5	0.327	±	0.089	0.238	±	0.087	0.023
Phosphatidylglycerol (PG)							
PG 34:2	10.7	±	2.8	7.87	±	1.34	0.004
PG 32:0	4.39	±	1.49	3.22	±	0.98	0.033
PG 34:1	51.8	±	9.4	41.7	±	6.6	0.006
Phosphatidylinositol (PI)							
PI 34:1	20.1	±	7.0	13.5	±	4.4	0.012
PI 36:3	10.1	±	2.7	7.95	±	1.61	0.023
Phosphatidylserine (PS)							

PS 38:3	12.1	±	2.6	9.83	±	1.29	0.007
PS 40:5	98.3	±	15.5	114	±	16	0.020
Sphingomyelin (SM)							
SM 36:1;O2	132	±	16	151	±	20	0.017

Shown are means and standard deviation (n = 12/group).

Table S3. qPCR validation of transcriptome data for selected differentially expressed transcripts (fold change (FC) > 1.3 or < −1.3, $p < 0.05$) in the *M. gastrocnemius* of adult male C57BL/6J mice. Mice were fed a diet containing either 0.3% ($P_{0.3}$) or 1.2% ($P_{1.2}$) phosphorus for 6 weeks.

Gene symbol	Gene Description	Transcriptome		qPCR	
		FC	*P*-value	FC	*P*-value
Chac1	ChaC. cation transport regulator 1	3.54	0.044	2.04	0.119
Gdap10	ganglioside-induced differentiation-associated-protein 10	1.95	0.022	-1.04	0.838
Lrp2bp	Lrp2 binding protein	1.57	0.035	1.15	0.421
Slc2a12	solute carrier family 2 (facilitated glucose transporter), member 12	1.48	0.001	1.35	0.041
Sfxn2	sideroflexin 2	1.44	0.001	1.21	0.207
Akt2	thymoma viral proto-oncogene 2	1.40	0.034	1.04	0.856
Tmem45b	transmembrane protein 45b	-1.52	0.011	-1.59	0.201
Fbxl12os	F-box and leucine-rich repeat protein 12, opposite strand	-1.60	0.016	-1.26	0.474
Mageb16	melanoma antigen family B, 16	-1.62	0.026	-1.46	0.424
Olfr1474	olfactory receptor 1474	-1.64	0.037	-1.34	0.488
Olfr384	olfactory receptor 384	-1.66	0.006	-1.61	0.229
Ric3	resistance to inhibitors of cholinesterase 3 homolog (C. elegans)	-1.67	0.013	-1.39	0.139

FC was calculated from n = 6 samples/group. qPCR FC was calculated from n = 12 samples/group.

Table S4. Fold change (FC) and *p*-values of all differentially expressed transcripts (FC > 1.3 or < −1.3, $p < 0.05$) in the *M. gastrocnemius* of adult male C57BL/6J mice. Mice were fed a diet containing either 0.3% ($P_{0.3}$) or 1.2% ($P_{1.2}$) phosphorus for 6 weeks.

Gene Symbol	Gene Description	FC	*P*-value
Chac1	ChaC, cation transport regulator 1	3.54	0.044
Arhgap26	Rho GTPase activating protein 26	2.26	0.037
Gdap10	ganglioside-induced differentiation-associated-protein 10	1.95	0.022
Clec4a2	C-type lectin domain family 4, member a2	1.86	0.005
Gm10600	predicted gene 10600 (Source:MGI Symbol;Acc:MGI:3710628)	1.70	0.011
Gm10490	predicted gene 10490 (Source:MGI Symbol;Acc:MGI:3642001)	1.66	0.028
Casp4	caspase 4, apoptosis-related cysteine peptidase	1.64	0.019
Prepl	prolyl endopeptidase-like	1.61	0.020
Ppm1k	protein phosphatase 1K (PP2C domain containing)	1.61	0.046
Pclo	piccolo (presynaptic cytomatrix protein)	1.60	0.007
Lce3a	late cornified envelope 3A	1.59	0.013
Lrp2bp	Lrp2 binding protein	1.57	0.035

Olfr1079	olfactory receptor 1079	1.55	0.012
Klhl38	kelch-like 38	1.54	0.038
Mchr1	melanin-concentrating hormone receptor 1	1.53	0.008
Polr1e	polymerase (RNA) I polypeptide E	1.51	0.012
Stbd1	starch binding domain 1	1.51	0.015
Gm11037	predicted gene 11037 (Source:MGI Symbol;Acc:MGI:3779261)	1.51	0.033
Abcb7	ATP-binding cassette, sub-family B (MDR/TAP), member 7	1.49	0.019
Foxj1	forkhead box J1	1.49	0.013
Isoc2a	isochorismatase domain containing 2a	1.49	0.045
Slc2a12	solute carrier family 2 (facilitated glucose transporter), member 12	1.48	0.001
Dync2h1	dynein cytoplasmic 2 heavy chain 1	1.47	0.007
Zfp369	zinc finger protein 369	1.47	0.025
Caml	calcium modulating ligand	1.46	0.012
Srbd1	S1 RNA binding domain 1	1.45	0.003
Gm9944	predicted gene 9944 (Source:MGI Symbol;Acc:MGI:3642412)	1.45	0.025
Prkra	protein kinase, interferon inducible double stranded RNA dependent activator	1.45	0.002
Gm11568	predicted gene 11568	1.45	0.005
Adgre5	adhesion g protein-coupled receptor E5	1.44	0.033
Cdh6	cadherin 6	1.44	0.017
4930572O03Rik	spermatogenesis associated glutamate (E)-rich protein pseudogene	1.44	0.013
Dnah6	dynein, axonemal, heavy chain 6	1.44	0.028
Sfxn2	sideroflexin 2	1.44	0.001
Nip7	nuclear import 7 homolog (S. cerevisiae)	1.43	0.007
Hist1h4d	histone cluster 1, H4d	1.43	0.022
Ampd2	adenosine monophosphate deaminase 2	1.43	0.009
Aldh4a1	aldehyde dehydrogenase 4 family, member A1	1.42	0.008
Mbnl3	muscleblind-like 3 (Drosophila)	1.42	0.036
4933408B17Rik	RIKEN cDNA 4933408B17 gene	1.42	0.007
Hscb	HscB iron-sulfur cluster co-chaperone homolog (E. coli)	1.42	0.040
Phf8	PHD finger protein 8	1.42	0.005
Mecr	mitochondrial trans-2-enoyl-CoA reductase	1.42	0.037
Tlr9	toll-like receptor 9	1.42	0.010
Olfr332	olfactory receptor 332	1.41	0.010
Snai3	snail family zinc finger 3	1.41	0.040
Pvrl3	poliovirus receptor-related 3	1.41	0.050
Gm8994	predicted gene 8994	1.41	0.037
Acot1	acyl-CoA thioesterase 1	1.41	0.012
Ddias	DNA damage-induced apoptosis suppressor	1.41	0.002
Pkhd1l1	polycystic kidney and hepatic disease 1-like 1	1.40	0.033
Trem2	triggering receptor expressed on myeloid cells 2	1.40	0.034
AI182371	expressed sequence AI182371	1.40	0.020
Akt2	thymoma viral proto-oncogene 2	1.40	0.034
Olfr832	olfactory receptor 832	1.40	0.043
Gm9930	predicted gene 9930 (Source:MGI Symbol;Acc:MGI:3642400)	1.40	0.021
Caap1	caspase activity and apoptosis inhibitor 1	1.40	0.008

Cyp3a59	cytochrome P450, family 3, subfamily a, polypeptide 59	1.40	0.013
Gm17615	predicted gene, 17615 (Source:MGI Symbol;Acc:MGI:4937249)	1.39	0.037
Gnpda1	glucosamine-6-phosphate deaminase 1	1.39	0.017
Acsl5	acyl-CoA synthetase long-chain family member 5	1.39	0.027
Peak1	pseudopodium-enriched atypical kinase 1	1.39	0.032
Aknad1	AKNA domain containing 1	1.39	0.049
Adhfe1	alcohol dehydrogenase, iron containing, 1	1.39	0.038
Mcm7	minichromosome maintenance deficient 7 (S. cerevisiae)	1.39	0.034
Tmie	transmembrane inner ear	1.39	0.024
Tyw3	tRNA-yW synthesizing protein 3 homolog (S. cerevisiae)	1.39	0.033
Edrf1	erythroid differentiation regulatory factor 1	1.39	0.022
Mtmr7	myotubularin related protein 7	1.39	0.002
Tas2r109	taste receptor, type 2, member 109	1.38	0.030
Car5b	carbonic anhydrase 5b, mitochondrial	1.38	0.035
Simc1	SUMO-interacting motifs containing 1	1.38	0.036
Zfp418	zinc finger protein 418	1.38	0.003
Ralgds	ral guanine nucleotide dissociation stimulator	1.38	0.032
Smpd2	sphingomyelin phosphodiesterase 2, neutral	1.38	0.039
1810037I17Rik	RIKEN cDNA 1810037I17 gene	1.38	0.000
Fgf2	fibroblast growth factor 2	1.38	0.015
Sycp1	synaptonemal complex protein 1	1.37	0.008
Kri1	KRI1 homolog (S. cerevisiae)	1.37	0.008
Adgrf4	adhesion g protein-coupled receptor F4	1.37	0.027
Paox	polyamine oxidase (exo-N4-amino)	1.37	0.023
Jag1	jagged 1	1.37	0.024
Trp53i13	transformation related protein 53 inducible protein 13	1.37	0.002
Saal1	serum amyloid A-like 1	1.37	0.011
Rsrp1	arginine/serine rich protein 1	1.37	0.046
Cpsf7	cleavage and polyadenylation specific factor 7	1.37	0.011
Olfr329-ps	olfactory receptor 329, pseudogene	1.37	0.018
Ercc5	excision repair cross-complementing rodent repair deficiency, complementation group 5	1.36	0.001
Cuedc1	CUE domain containing 1	1.36	0.037
Apex1	apurinic/apyrimidinic endonuclease 1	1.36	0.003
Rbmxl1	RNA binding motif protein, X-linked like-1	1.36	0.021
Trip13	thyroid hormone receptor interactor 13	1.36	0.010
Apobec3	apolipoprotein B mRNA editing enzyme, catalytic polypeptide 3	1.35	0.032
Olfr1216	olfactory receptor 1216	1.35	0.047
Gabrg3	gamma-aminobutyric acid (GABA) A receptor, subunit gamma 3	1.35	0.040
Ints3	integrator complex subunit 3	1.35	0.002
Golgb1	golgi autoantigen, golgin subfamily b, macrogolgin 1	1.35	0.003
Gm3002	alpha-takusan pseudogene	1.35	0.040
Gabra6	gamma-aminobutyric acid (GABA) A receptor, subunit alpha 6	1.35	0.050
Nepro	nucleolus and neural progenitor protein	1.35	0.045
1700007K13Rik	RIKEN cDNA 1700007K13 gene	1.34	0.050
Nup205	nucleoporin 205	1.34	0.005

Tusc3	tumor suppressor candidate 3	1.34	0.007
Frmd4b	FERM domain containing 4B	1.34	0.001
P2rx4	purinergic receptor P2X, ligand-gated ion channel 4	1.33	0.043
Ankrd35	ankyrin repeat domain 35	1.33	0.042
Olfr1109	olfactory receptor 1109	1.33	0.033
Ppm1e	protein phosphatase 1E (PP2C domain containing)	1.33	0.000
Tmlhe	trimethyllysine hydroxylase, epsilon	1.33	0.034
Fam213b	family with sequence similarity 213, member B	1.33	0.017
Stard7	START domain containing 7	1.33	0.015
Magee1	melanoma antigen, family E, 1	1.33	0.046
Dna2	DNA replication helicase 2 homolog (yeast)	1.33	0.011
Gm2483	PREDICTED: predicted gene 2483 (Gm2483), mRNA.	1.33	0.019
Pccb	propionyl Coenzyme A carboxylase, beta polypeptide	1.32	0.027
Pik3ip1	phosphoinositide-3-kinase interacting protein 1	1.32	0.008
Slc24a2	solute carrier family 24 (sodium/potassium/calcium exchanger), member 2	1.32	0.016
Gadd45a	growth arrest and DNA-damage-inducible 45 alpha	1.32	0.040
E2f3	E2F transcription factor 3	1.32	0.036
Gm21788	predicted gene, 21788 (Source:MGI Symbol;Acc:MGI:5433952)	1.32	0.025
Tspo	translocator protein	1.32	0.021
Smarcd2	SWI/SNF related, matrix associated, actin dependent regulator of chromatin, subfamily d, member 2	1.32	0.014
Cd300a	CD300A antigen	1.32	0.029
Dtx3l	deltex 3-like (Drosophila)	1.32	0.007
Cd300c	CD300C antigen	1.32	0.034
Dgke	diacylglycerol kinase, epsilon	1.32	0.021
Ppp2r2b	protein phosphatase 2, regulatory subunit B, beta	1.32	0.044
Tmem185b	transmembrane protein 185B	1.32	0.048
Arhgef10	Rho guanine nucleotide exchange factor (GEF) 10	1.31	0.028
Suv39h2	suppressor of variegation 3-9 homolog 2 (Drosophila)	1.31	0.039
Il23a	interleukin 23, alpha subunit p19	1.31	0.030
H2-T22	histocompatibility 2, T region locus 22	1.31	0.031
A230083G16Rik	RIKEN cDNA A230083G16 gene	1.31	0.029
Olfr733	olfactory receptor 733	1.30	0.024
Rnf225	ring finger protein 225	1.30	0.022
C5ar1	complement component 5a receptor 1	1.30	0.023
Fbxw27	PREDICTED: F-box and WD-40 domain protein 27, transcript variant 1 (Fbxw27), mRNA.	1.30	0.008
Icosl	icos ligand	1.30	0.009
Spryd3	SPRY domain containing 3	1.30	0.037
Ptp4a3	protein tyrosine phosphatase 4a3	1.30	0.004
Ctu1	cytosolic thiouridylase subunit 1 homolog (S. pombe)	1.30	0.048
Magea4	melanoma antigen, family A, 4	1.30	0.018
1700030K09Rik	RIKEN cDNA 1700030K09 gene	-1.30	0.034
Ppp2r2c	protein phosphatase 2, regulatory subunit B, gamma	-1.30	0.032
1700066B19Rik	RIKEN cDNA 1700066B19 gene	-1.30	0.007

Akr1c19	aldo-keto reductase family 1, member C19	-1.30	0.013
Pnma3	paraneoplastic antigen MA3	-1.30	0.036
Stx19	syntaxin 19	-1.30	0.033
Adgre4	adhesion g protein-coupled receptor E4	-1.30	0.025
Unc79	unc-79 homolog (C. elegans)	-1.30	0.018
Gm10573	PREDICTED: predicted gene 10573 (Gm10573), mRNA.	-1.30	0.008
Spata32	spermatogenesis associated 32	-1.31	0.007
Zyx	zyxin	-1.31	0.023
Slc22a29	solute carrier family 22. member 29	-1.31	0.022
Olfr159	olfactory receptor 159	-1.31	0.024
1500015O10Rik	RIKEN cDNA 1500015O10 gene	-1.31	0.031
Fancm	Fanconi anemia, complementation group M	-1.31	0.017
Rhox4f	reproductive homeobox 4F	-1.31	0.012
Olfr802	olfactory receptor 802	-1.31	0.023
Prl3a1	prolactin family 3, subfamily a, member 1	-1.31	0.040
Strip2	striatin interacting protein 2	-1.31	0.014
Creb3l3	cAMP responsive element binding protein 3-like 3	-1.31	0.024
Itga2	integrin alpha 2	-1.31	0.009
Ikzf3	IKAROS family zinc finger 3	-1.31	0.000
Csf3r	colony stimulating factor 3 receptor (granulocyte)	-1.31	0.045
Scgb1a1	secretoglobin, family 1A, member 1 (uteroglobin)	-1.31	0.013
Ccdc171	coiled-coil domain containing 171	-1.31	0.007
LOC100862019	PREDICTED: palmitoyl-protein thioesterase 1-like (LOC100862019), mRNA.	-1.31	0.002
Diras1	DIRAS family, GTP-binding RAS-like 1	-1.32	0.021
Gm20736	predicted gene, 20736	-1.32	0.029
Gm11562	predicted gene 11562	-1.32	0.012
Hist1h2aa	histone cluster 1, H2aa	-1.32	0.012
Srgap1	SLIT-ROBO Rho GTPase activating protein 1	-1.32	0.012
Tmc1	transmembrane channel-like gene family 1	-1.32	0.005
Btnl5-ps	butyrophilin-like 5, pseudogene	-1.32	0.025
Olfr513	olfactory receptor 513	-1.32	0.034
Mrgprf	MAS-related GPR, member F	-1.32	0.002
Fga	fibrinogen alpha chain	-1.32	0.045
Speer4f2	spermatogenesis associated glutamate (E)-rich protein 4f2 (Source:MGI Symbol;Acc:MGI:3781672)	-1.32	0.038
Col6a4	collagen, type VI, alpha 4	-1.32	0.001
Gm7233	predicted gene 7233 (Source:MGI Symbol;Acc:MGI:3648176)	-1.32	0.038
Btbd16	BTB (POZ) domain containing 16	-1.32	0.007
Gm16405	predicted gene 16405	-1.32	0.029
Gm16405	predicted gene 16405	-1.32	0.029
Ceacam18	carcinoembryonic antigen-related cell adhesion molecule 18	-1.32	0.025
Adam1b	a disintegrin and metallopeptidase domain 1b	-1.32	0.021
1110017D15Rik	RIKEN cDNA 1110017D15 gene	-1.32	0.049
Cpne2	copine II	-1.32	0.028
Slc5a7	solute carrier family 5 (choline transporter), member 7	-1.33	0.035

Vmn1r129	vomeronasal 1 receptor 129	-1.33	0.032
Cyp2c70	cytochrome P450, family 2, subfamily c, polypeptide 70	-1.33	0.035
A930018P22Rik	RIKEN cDNA A930018P22 gene	-1.33	0.001
Sstr5	somatostatin receptor 5	-1.33	0.005
Cd209c	CD209c antigen	-1.33	0.030
Thsd7a	thrombospondin, type I, domain containing 7A	-1.33	0.049
Timd4	T cell immunoglobulin and mucin domain containing 4	-1.33	0.033
Fam19a4	family with sequence similarity 19, member A4	-1.33	0.049
Fpr1	formyl peptide receptor 1	-1.33	0.044
Traf3	TNF receptor-associated factor 3	-1.33	0.019
Nppc	natriuretic peptide type C	-1.33	0.010
Gm17714	PREDICTED: predicted gene, 17714 (Gm17714), mRNA.	-1.33	0.005
Gm10230	predicted gene 10230	-1.34	0.023
Gm6455	predicted gene 6455	-1.34	0.025
Tas2r143	taste receptor, type 2, member 143	-1.34	0.042
Prdm6	PR domain containing 6	-1.34	0.007
Gm10486	predicted gene 10486	-1.34	0.039
Olfml1	olfactomedin-like 1	-1.34	0.028
Baiap2l2	BAI1-associated protein 2-like 2	-1.34	0.034
Unc5b	unc-5 homolog B (C. elegans)	-1.34	0.013
Phgr1	proline/histidine/glycine-rich 1	-1.34	0.022
Adgrg7	adhesion g protein-coupled receptor G7	-1.34	0.038
Trpc5	transient receptor potential cation channel, subfamily C, member 5	-1.34	0.018
Spc25	SPC25, NDC80 kinetochore complex component, homolog (S. cerevisiae)	-1.34	0.030
Spata3	spermatogenesis associated 3	-1.34	0.003
Gpr87	G protein-coupled receptor 87	-1.35	0.003
Cdrt4	CMT1A duplicated region transcript 4	-1.35	0.026
Sh2d4b	SH2 domain containing 4B	-1.35	0.030
Olfr107	olfactory receptor 107	-1.35	0.030
Gabrb3	gamma-aminobutyric acid (GABA) A receptor, subunit beta 3	-1.35	0.043
Gemin6	gem (nuclear organelle) associated protein 6	-1.35	0.018
Olfr1449	olfactory receptor 1449	-1.35	0.034
Cyp2t4	cytochrome P450, family 2, subfamily t, polypeptide 4	-1.35	0.048
Fbxo27	F-box protein 27	-1.35	0.023
Gm20888	predicted gene, 20888 (Source:MGI Symbol;Acc:MGI:5434244)	-1.35	0.048
Timeless	timeless circadian clock 1	-1.35	0.013
Gm11564	predicted gene 11564	-1.35	0.001
Cdh20	cadherin 20	-1.36	0.012
Fam110c	family with sequence similarity 110, member C	-1.36	0.015
Olfr715	olfactory receptor 715	-1.36	0.036
Defb29	defensin beta 29	-1.36	0.002
Vmn1r79	vomeronasal 1 receptor 79	-1.36	0.024
Smim1	small integral membrane protein 1	-1.36	0.010
Gm9696	arylacetamide deacetylase-like 2 pseudogene	-1.36	0.041
Gm6377	predicted gene 6377	-1.36	0.033
Gm15128	predicted gene 15128	-1.36	0.003

Olfr221	olfactory receptor 221	-1.37	0.003
Tbxa2r	thromboxane A2 receptor	-1.37	0.023
Fgf21	fibroblast growth factor 21	-1.37	0.035
Nin	ninein	-1.37	0.036
Vmn1r33	vomeronasal 1 receptor 33	-1.37	0.033
Olfr730	olfactory receptor 730	-1.37	0.041
Cnrip1	cannabinoid receptor interacting protein 1	-1.37	0.036
Msantd1	Myb/SANT-like DNA-binding domain containing 1	-1.37	0.017
Olfr410	olfactory receptor 410	-1.37	0.010
Olfr1215	olfactory receptor 1215	-1.37	0.029
Gzmd	granzyme D	-1.37	0.036
Gm20937	predicted gene, 20937 (Source:MGI Symbol;Acc:MGI:5434293)	-1.38	0.012
Gabra3	gamma-aminobutyric acid (GABA) A receptor, subunit alpha 3	-1.38	0.035
S100g	S100 calcium binding protein g	-1.38	0.039
Chil1	chitinase-like 1	-1.38	0.010
Dsc1	desmocollin 1	-1.38	0.011
Gpr65	G-protein coupled receptor 65	-1.38	0.039
Gm9513	predicted gene 9513	-1.38	0.041
Gucy1a2	guanylate cyclase 1, soluble, alpha 2	-1.38	0.040
Gm8890	predicted gene 8890 (Source:MGI Symbol;Acc:MGI:3779817)	-1.38	0.045
Tmem145	transmembrane protein 145	-1.38	0.017
Slc24a1	solute carrier family 24 (sodium/potassium/calcium exchanger), member 1	-1.38	0.003
Gm17449	predicted gene, 17449 (Source:MGI Symbol;Acc:MGI:4937083)	-1.38	0.017
LOC100040054	PREDICTED: X-linked lymphocyte-regulated protein PM1-like, transcript variant 1 (LOC100040054)	-1.38	0.008
Vmn2r86	vomeronasal 2, receptor 86	-1.39	0.033
St8sia4	ST8 alpha-N-acetyl-neuraminide alpha-2,8-sialyltransferase 4	-1.39	0.004
Awat1	acyl-CoA wax alcohol acyltransferase 1	-1.39	0.030
Kifc3	kinesin family member C3	-1.39	0.012
Osgin1	oxidative stress induced growth inhibitor 1	-1.40	0.016
Shf	Src homology 2 domain containing F	-1.40	0.013
Gck	glucokinase	-1.40	0.002
Bpifb9a	BPI fold containing family B, member 9A	-1.40	0.035
Zfp648	zinc finger protein 648	-1.40	0.004
Gm10486	predicted gene 10486	-1.40	0.022
Sult2a1	sulfotransferase family 2A, dehydroepiandrosterone (DHEA)-preferring, member 1	-1.40	0.022
Tacr1	tachykinin receptor 1	-1.40	0.011
Rgs18	regulator of G-protein signaling 18	-1.40	0.013
Ccdc25	coiled-coil domain containing 25	-1.40	0.046
Tgif2lx2	TGFB-induced factor homeobox 2-like, X-linked 2	-1.41	0.038
Hapln3	hyaluronan and proteoglycan link protein 3	-1.41	0.012
Cd200r2	Cd200 receptor 2	-1.41	0.015
Sema3e	sema domain, immunoglobulin domain (Ig), short basic domain, secreted, (semaphorin) 3E	-1.41	0.026
Vmn2r118	vomeronasal 2, receptor 118	-1.41	0.016

Olfr765	olfactory receptor 765	-1.41	0.018
Klhl5	kelch-like 5	-1.41	0.003
4921504E06Rik	RIKEN cDNA 4921504E06 gene	-1.41	0.025
Obp2a	odorant binding protein 2A	-1.41	0.030
Esp16	exocrine gland secreted peptide 16	-1.41	0.003
Fntb	farnesyltransferase, CAAX box, beta	-1.41	0.023
Vmn2r22	vomeronasal 2, receptor 22	-1.41	0.003
1700012B07Rik	RIKEN cDNA 1700012B07 gene	-1.41	0.001
Olfr1328	olfactory receptor 1328	-1.41	0.029
Olfr1086	olfactory receptor 1086	-1.41	0.032
Efcab10	EF-hand calcium binding domain 10	-1.41	0.021
Patl2	protein associated with topoisomerase II homolog 2 (yeast)	-1.41	0.006
Vmn1r169	vomeronasal 1 receptor 169	-1.41	0.027
Vmn1r18	vomeronasal 1 receptor 18	-1.42	0.018
Pask	PAS domain containing serine/threonine kinase	-1.42	0.023
Zfp354b	zinc finger protein 354B	-1.42	0.010
B3glct	beta-3-glucosyltransferase	-1.42	0.014
Gm16405	predicted gene 16405	-1.42	0.031
Mrgpra1	MAS-related GPR, member A1	-1.42	0.004
4933427E11Rik	RIKEN cDNA 4933427E11 gene	-1.42	0.001
Sprr1b	small proline-rich protein 1B	-1.42	0.040
Gp1ba	glycoprotein 1b, alpha polypeptide	-1.42	0.038
Olfr385	olfactory receptor 385	-1.42	0.041
Vmn2r27	vomeronasal 2, receptor27	-1.43	0.043
Lrriq4	leucine-rich repeats and IQ motif containing 4	-1.43	0.014
Spata31d1a	spermatogenesis associated 31 subfamily D, member 1A	-1.43	0.008
Wfdc17	WAP four-disulfide core domain 17	-1.43	0.009
Cdh7	cadherin 7, type 2	-1.43	0.010
Prtg	protogenin homolog (Gallus gallus)	-1.43	0.036
Olfr725	olfactory receptor 725	-1.43	0.022
Fcer2a	Fc receptor, IgE, low affinity II, alpha polypeptide	-1.43	0.014
Slc1a7	solute carrier family 1 (glutamate transporter), member 7	-1.44	0.004
BC048671	cDNA sequence BC048671	-1.44	0.003
Hivep3	human immunodeficiency virus type I enhancer binding protein 3	-1.44	0.030
Olfr924	olfactory receptor 924	-1.44	0.010
Adam6a	a disintegrin and metallopeptidase domain 6A	-1.44	0.021
Rgs5	regulator of G-protein signaling 5	-1.44	0.032
Tmem253	transmembrane protein 253	-1.44	0.014
Ear7	eosinophil-associated, ribonuclease A family, member 7	-1.44	0.022
Tmem169	transmembrane protein 169	-1.45	0.006
Olfr1283	olfactory receptor 1283	-1.45	0.031
Olfr549	olfactory receptor 549	-1.45	0.013
Ces3a	carboxylesterase 3A	-1.45	0.038
2610524H06Rik	RIKEN cDNA 2610524H06 gene	-1.45	0.017
4930595M18Rik	RIKEN cDNA 4930595M18 gene	-1.46	0.044
1700008O03Rik	RIKEN cDNA 1700008O03 gene	-1.46	0.003

Pmel	premelanosome protein	-1.47	0.033
Gm16493	predicted gene 16493	-1.47	0.005
Cfap73	cilia and flagella associated protein 73	-1.47	0.031
Lep	leptin	-1.47	0.006
Olfr1294	olfactory receptor 1294	-1.47	0.036
Cyp3a44	cytochrome P450, family 3, subfamily a, polypeptide 44	-1.47	0.015
Tnn	tenascin N	-1.47	0.013
Lpar2	lysophosphatidic acid receptor 2	-1.48	0.006
Esrp2	epithelial splicing regulatory protein 2	-1.48	0.007
Slc39a12	solute carrier family 39 (zinc transporter), member 12	-1.48	0.025
Pgf	placental growth factor	-1.48	0.033
Kcnmb2	potassium large conductance calcium-activated channel, subfamily M, beta member 2	-1.49	0.006
Lgi2	leucine-rich repeat LGI family, member 2	-1.49	0.044
Gm15299	predicted pseudogene 15299	-1.49	0.026
Rab31	RAB31, member RAS oncogene family	-1.49	0.034
Gm8922	predicted gene 8922 (Source:MGI Symbol;Acc:MGI:3779821)	-1.50	0.018
Olfr1276	olfactory receptor 1276	-1.50	0.012
LOC100045026	butyrophilin-like	-1.51	0.016
Gm10283	predicted gene 10283 (Source:MGI Symbol;Acc:MGI:3647628)	-1.51	0.025
Serpinb9b	serine (or cysteine) peptidase inhibitor, clade B, member 9b	-1.51	0.004
Lyzl6	lysozyme-like 6	-1.51	0.006
Btg1-ps1	B cell translocation gene 1, anti-proliferative, pseudogene 1	-1.52	0.022
Tmem45b	transmembrane protein 45b	-1.52	0.011
Vmn1r167	vomeronasal 1 receptor 167	-1.52	0.008
Gm21907	predicted gene, 21907	-1.52	0.001
Gm21915	predicted gene, 21915 (Source:MGI Symbol;Acc:MGI:5434079)	-1.53	0.016
Olfr958	olfactory receptor 958	-1.53	0.049
Mrc2	mannose receptor, C type 2	-1.54	0.025
Lrrc17	leucine rich repeat containing 17	-1.54	0.011
Cntn5	contactin 5	-1.56	0.007
Gm8653	predicted gene 8653	-1.57	0.035
Lst1	leukocyte specific transcript 1	-1.58	0.004
Olfr141	olfactory receptor 141	-1.60	0.003
Fbxl12os	F-box and leucine-rich repeat protein 12, opposite strand	-1.60	0.016
Mageb16	melanoma antigen family B, 16	-1.62	0.026
Olfr1474	olfactory receptor 1474	-1.64	0.037
Olfr384	olfactory receptor 384	-1.66	0.006
Ric3	resistance to inhibitors of cholinesterase 3 homolog (C. elegans)	-1.67	0.013
Lipi	lipase, member I	-1.71	0.002

FC was calculated from n = 6 microarrays/group
